# Acute Multiple Organ Failure in Adult Mice Deleted for the Developmental Regulator Wt1

**DOI:** 10.1371/journal.pgen.1002404

**Published:** 2011-12-22

**Authors:** You-Ying Chau, David Brownstein, Heidi Mjoseng, Wen-Chin Lee, Natalija Buza-Vidas, Claus Nerlov, Sten Eirik Jacobsen, Paul Perry, Rachel Berry, Anna Thornburn, David Sexton, Nik Morton, Peter Hohenstein, Elisabeth Freyer, Kay Samuel, Rob van't Hof, Nicholas Hastie

**Affiliations:** 1 Medical Research Council Human Genetics Unit and the Institute of Genetics and Molecular Medicine, Western General Hospital, Edinburgh, United Kingdom; 2 Queen's Medical Research Institute, Edinburgh, United Kingdom; 3 Centre for Regenerative Medicine, University of Edinburgh, Edinburgh, United Kingdom; 4 Division of Nephrology, Department of Internal Medicine, Kaohsiung Chang Gung Memorial Hospital and Chang Gung University College of Medicine, Kaohsiung, Taiwan; 5 Institute of Stem Cell Research, Medical Research Council Centre for Regenerative Medicine, Edinburgh, United Kingdom; 6 The Weatherall Institute of Molecular Medicine, John Radcliffe Hospital, University of Oxford, Oxford, United Kingdom; 7 Centre for Cardiovascular Science, University of Edinburgh, Edinburgh, United Kingdom; 8 Scottish National Blood Transfusion Service, Centre for Regenerative Medicine, Edinburgh, United Kingdom; 9 Molecular Medicine Centre and the Institute of Genetics and Molecular Medicine, Western General Hospital, Edinburgh, United Kingdom; University of Oxford, United Kingdom

## Abstract

There is much interest in the mechanisms that regulate adult tissue homeostasis and their relationship to processes governing foetal development. Mice deleted for the Wilms' tumour gene, *Wt1*, lack kidneys, gonads, and spleen and die at mid-gestation due to defective coronary vasculature. Wt1 is vital for maintaining the mesenchymal–epithelial balance in these tissues and is required for the epithelial-to-mesenchyme transition (EMT) that generates coronary vascular progenitors. Although Wt1 is only expressed in rare cell populations in adults including glomerular podocytes, 1% of bone marrow cells, and mesothelium, we hypothesised that this might be important for homeostasis of adult tissues; hence, we deleted the gene ubiquitously in young and adult mice. Within just a few days, the mice suffered glomerulosclerosis, atrophy of the exocrine pancreas and spleen, severe reduction in bone and fat, and failure of erythropoiesis. FACS and culture experiments showed that Wt1 has an intrinsic role in both haematopoietic and mesenchymal stem cell lineages and suggest that defects within these contribute to the phenotypes we observe. We propose that glomerulosclerosis arises in part through down regulation of nephrin, a known Wt1 target gene. Protein profiling in mutant serum showed that there was no systemic inflammatory or nutritional response in the mutant mice. However, there was a dramatic reduction in circulating IGF-1 levels, which is likely to contribute to the bone and fat phenotypes. The reduction of IGF-1 did not result from a decrease in circulating GH, and there is no apparent pathology of the pituitary and adrenal glands. These findings 1) suggest that Wt1 is a major regulator of the homeostasis of some adult tissues, through both local and systemic actions; 2) highlight the differences between foetal and adult tissue regulation; 3) point to the importance of adult mesenchyme in tissue turnover.

## Author Summary

It is important to understand the cellular and molecular pathways that regulate the maintenance and turnover of adult tissues. These processes often go awry in diseases and are likely to deteriorate with ageing. Here we show that removal of a single gene, the Wilms' Tumour gene, *Wt1*, in the adult mouse leads to the extremely rapid deterioration of multiple tissues. Within 7–9 days after gene removal kidneys fail, the pancreas and spleen suffer severe atrophy, there is widespread loss of bone and body fat, and red blood cells are no longer produced. Our findings reveal the vulnerability of adult tissues, while opening up avenues for dissecting the pathways controlling tissue turnover. Further experiments showed that the tissue failure we observed is due both to local defects of stem/progenitor cell activities and to significant changes in the serum levels of some key master regulators. In particular there is a dramatic reduction in the levels of IGF-1, a key regulator of homeostasis and aging. Our studies also show that the control of adult tissue turnover may be different from that during foetal development. These findings have important implications for understanding and treating common human diseases.

## Introduction

Although much is known about the mechanisms that govern cellular differentiation during development, we know less about the processes that regulate cell turnover and homeostasis in the adult. Perhaps the exceptions to this rule are rapidly turning over tissues such as intestine, skin and haematopoietic tissue. Recently it has been shown that genes required for regulating differentiation during foetal development may not be used in regulating turnover of the same tissues in the adult [1], [2].

Mutation of the Wilms tumour gene, *WT1*, in humans may lead to the eponymous paediatric kidney cancer, glomerulosclerosis of the kidney and gonadal dysgenesis, which can manifest as male to female sex reversal [3]. During foetal development, Wt1 is expressed in the kidney, gonads, spleen, the mesothelium which surrounds most organs as well as ill-defined body mesenchyme. Knockout mice lack kidneys, gonads, and spleen and the animals die at mid-gestation through the lack of coronary vasculature formation [4]. There are no apparent defects of the skeletal, haematopoietic, digestive, or metabolic systems.

Recently we have shown that Wt1 is a key regulator of the balance between the epithelial and mesenchymal states in a number of developing organs. Whereas it is required for the mesenchymal to epithelial transition (MET) underlying the formation of kidney nephrons, in the heart it is essential for the reverse process, the epithelial to mesenchyme transition (EMT) required for the production of proliferating cardiovascular progenitors from the epicardium (a mesothelium) [5]. In a similar vein Wt1 expressing mesothelial cells in the intestine and lung produce mesenchymal progenitors for vascular smooth muscle [6], [7]. Furthermore, very recent evidence proves that, in the developing liver, Wt1 expressing mesothelial cells provide the precursors for stellate cells [8], [9], [10]. Stellate cells in the liver and the pancreas have aroused much interest through their ability to regulate tissue fibrosis, via the production of cytokines [11], [12]. They are also important for the progression of pancreatic cancer [13].

In the adult, Wt1 is expressed in very few tissues in a small percent of cells. These include the mesothelium surrounding a number of visceral organs [14], the glomerular podocyte cells of the kidney, Sertoli/granulosa cells in the testes/ovaries [15], [16], [17] and 1% of bone marrow (BM) cells (with properties of restricted haematopoietic progenitors) [18]. Nothern Blot analysis has shown that Wt1 is also expressed in a variety of epithelial cells including spleen, lung and heart. Our own data, including those provided in this paper suggest that this mainly reflects expression in the mesothelial lining of these tissues. We speculated that the expression of Wt1 in these rare sites in the adult could have functional significance, for the following reasons. Firstly, given the importance of the mesothelium as a source of progenitor cells, requiring Wt1 function during development, we hypothesised that mesothelia might perform a similar function in the adult and this might require Wt1.

Secondly, Wt1 is essential for the formation and maturation of podocytes [19]. We hypothesised that continued expression of Wt1 in the adult would reflect a role in kidney maintenance.

Thirdly, *WT1* is mutated or overexpressed in acute myeloid leukaemia (AML) [20]. However, Wt1 is not required for foetal haematopoiesis [21]. Given Wt1 expression in adult bone marrow and association with leukaemia, we surmised that Wt1 might play a role in adult haematopoiesis.

Finally, WT1 is expressed at high levels in most adult cancers studied [22], though expression has not been detected in the normal tissue counterparts. It has been proposed that WT1 might be an oncogene in adult cancer in contrast to its function as a tumour suppressor in paediatric kidney cancer [3]. As a prelude to testing this, it was necessary first to determine whether the gene is essential for normal development or maintenance of the epithelia from which these tumours arise.

To address these propositions, we deleted the *Wt1* gene ubiquitously in adult mice. While our findings inform on these issues, the results far exceeded our expectations. The range, severity, and rapidity of the phenotypes observed were dramatic and unexpected and raise major questions about adult tissue homeostasis.

## Results

### Tamoxifen-mediated deletion of *Wt1*

To enable inducible deletion of *Wt1* in the adult, we generated tamoxifen inducible *Wt1* KOs by crossing *CAGG* promoter driven Cre-ER™ mice with our homozygous *Wt1* conditional mice, where the first exon of *Wt1* is flanked by *loxP* sites [5]. Successful *Wt1* deletion was demonstrated by recombination PCR and the depletion of Wt1 expression in mesothelia (Figure S1 and Figure S2). Deletion of Wt1 in the mesothelium did not affect the integrity of the tissue (Figure S3). The health status of the mutant animals deteriorated quickly and all the mice had to be culled by 10 days post-induction (p.i.). Prior to death, the mutant mice presented dramatic phenotypes; they were less active and oedemic. Upon dissection, fluid was sometimes found in the abdominal cavity and in the subcutaneous tissues. Detailed gravimetric analysis showed that there was a reduction in the spleen to body weight ratio as well as in the heart to body weight ratio (Table 1). Subsequent histological analysis of internal organs revealed pale kidneys, severe spleen and pancreas atrophy, and deficiency of fat tissues. For most tissues, mice treated at 3, 10, or 13 weeks of age developed the same phenotypes. The only exception to this involved fat, as we discuss in more detail later. Before considering each phenotype, it is important to emphasise that not all tissues showed overt signs of damage. For example, we observed no obvious macroscopic changes to the lung, liver or intestine- three tissues often involved in systemic inflammatory responses. Furthermore, although there was a 30% reduction in the heart/body weight ratio there was no obvious cardiovascular pathology (Table 1).

**Table 1:** Summary of the gravemetrics of adult mice deleted for Wt1.

Mature mice						
	Weight	Spleen/BW %	Kidney/BW %	Heart/BW %	Liver/BW %	Testes/BW %
Mutant	20.07	0.260±0.026	1.673±0.085	0.509±0.027	7.103±0.55	0.640±0.60
		n = 9	n = 9	n = 9	n = 9	n = 6
Control	20.87	0.586±0.033	1.548±0.053	0.714±0.093	6.780±0.34	0.793±0.053
		n = 12	n = 9	n = 10	n = 11	n = 7
*P*-value	0.563	0.000^*^	0.239	0.003^*^	0.849	0.073

### Deletion of *Wt1* leads to acute glomerulosclerosis

**Figure 1:**
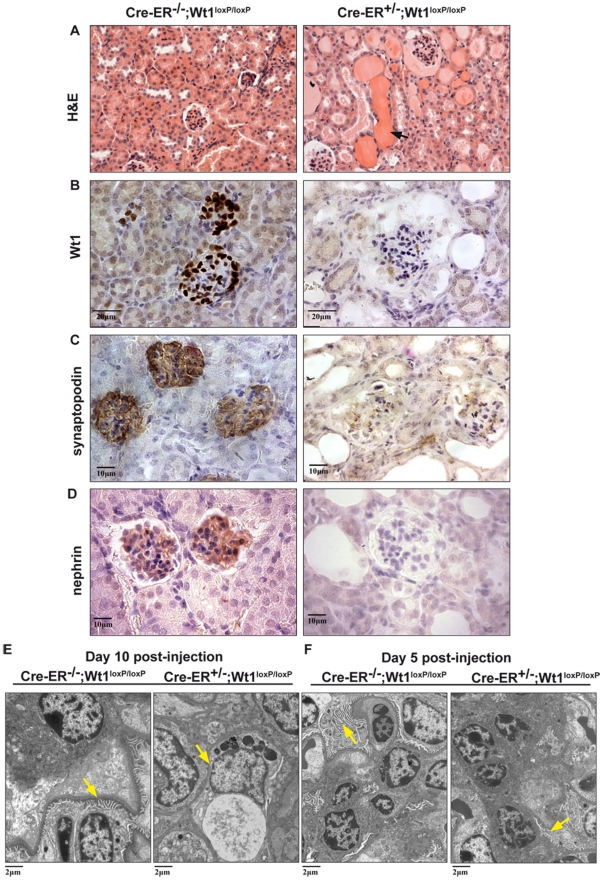
Severe kidney phenotype in the adult conditional *Wt1* KOs. A, At day 10 post-injection, H&E staining of paraffin sections showing accumulation of protein cast in the mutant kidney (right) c.f. control (left). B, Kidney sections stained with a Wt1-specific antibody in the mutant kidney (right) c.f. control (left); scale bar, 20 µm. C, Immunostaining of a podocyte marker synaptopodin; scale bar, 10 µm. D, Immunostaining of nephrin in the control and mutant kidney (right); scale bar, 10 µm. E, EM studies show the presence of foot process (arrows) of the podocytes in control mice (left) while the foot process is completely abolished in the mutants (right) at day 10 postinjection. F, At day 5 post-injection, effacement of foot process starts to show in the mutants c.f. the normal controls; scale bar, 2 µm.

Wt1 is crucial for kidney development as the conventional *Wt1*-null embryos suffer from renal agenesis [4]. Upon induction of Wt1 deletion in our model, expression of Wt1 in the podocytes was completely depleted (Figure 1B) and the mutant mice were shown to have severe proteinuria (Table 2). H&E staining showed that the tubules were filled with protein casts (Figure 1A, arrow). The mutant kidneys had well developed glomerulopathy with cytopathic changes in podocytes and parietal epithelium. There was almost complete loss of synaptopodin and nephrin expression in the podocytes in the mutant kidneys (Figure 1C and 1D). EM studies showed that the foot processes of the podocytes were completely lost in the mutant kidneys (Figure 1E, day 10 post-injection). The development of the kidney phenotype in our model was extremely rapid. Five days post-tamoxifen injection, H&E stained kidney sections showed normal histology while podocyte effacement started to appear (Figure 1F). At day 7 post-injection, protein casts in the tubules were already present and the glomeruli started showing signs of degeneration (Figure S4a). Finally, plasma levels of urea and creatinine were normal at day 5 p.i., started to rise at day 7 p.i., and were significantly elevated at day 10 p.i (Table 2). In our model, mice that were heterozygous for the *Wt1* conditional allele (*CAGG-CreER*™; *Wt1* ^loxP/+^) did not exhibit any kidney abnormalities after tamoxifen-mediated deletion of *Wt1*. In addition, tamoxifen treated mice that were only positive for the *CAGG-CreER*™ allele and wild type for the *Wt1 loxP* sites (i.e. *CAGG-CreER*™ positive; *Wt1* ^+/+^) were also included as controls and did not demonstrate any phenotypes. The kidney phenotype in our model is similar to other nephrotic syndrome mice where podocytes are damaged [23], [24], [25], [26]. However, none of these other mouse models presented any of the other phenotypes we describe below apart from the kidney defects. Most importantly, Wt1 has been deleted specifically in adult podocytes. These animals develop glomeruloscelosis similar to that described here but did not develop the other phenotypes we report below. Furthermore, the mice survived well beyond the timeframe reported here [27].

**Table 2:** Urine and serum biochemistry analysis of adult mice deleted for Wt1.

		N	Urine protein (mg/dl)	Serum urea (mmol/l)	Serum Creatinine (mol/l)	Serum albumin (g/l)	Serum amylase (U/l)
Day 5	Control	4		5±1.81	9.63±1.13	25.98±1.78	560.7±64.7
	Mutant	3		4.6±0.26	8.1±1.56	22.4±2.55	597.3±41.5
	P-value			0.857	0.533	0.267	0.533
Day 7	Control	6		5.62±0.88	11.82±1.08	21.42±2.23	427.3±96.3
	Mutant	5		11.08±4.22	15.25±3.51	Not detected	367±34.6
	P-value			0.021*	0.127	n/a	0.289
Day 10	Control	5	65.1±15.2	6.66±0.78	12.08±0.76	25.85±1.27	648±117
	Mutant	3	1503.7±137.3	30.17±8.47	58.6±51.33	15.4±0.57	392±172
	*P*-value		0.05*	0.025*	0.064	0.083	0.165

Wt1 is expressed at E9 in the urogenital ridge and subsequently in the sex cords of the genital ridge in mice and it is a crucial factor for gonad development and sex determination [28]. In adult mice, Wt1 is expressed in Sertoli cells in the testes and granulosa cells in the ovaries [15]. We observed a reduction in the size of the testes and ovaries; however the difference was not statistically significant (Table 1). None of the testis markers studied showed any difference in expression patterns (Figure S5).

### Deletion of *Wt1* leads to an aberrant haematopoietic system

**Figure 2:**
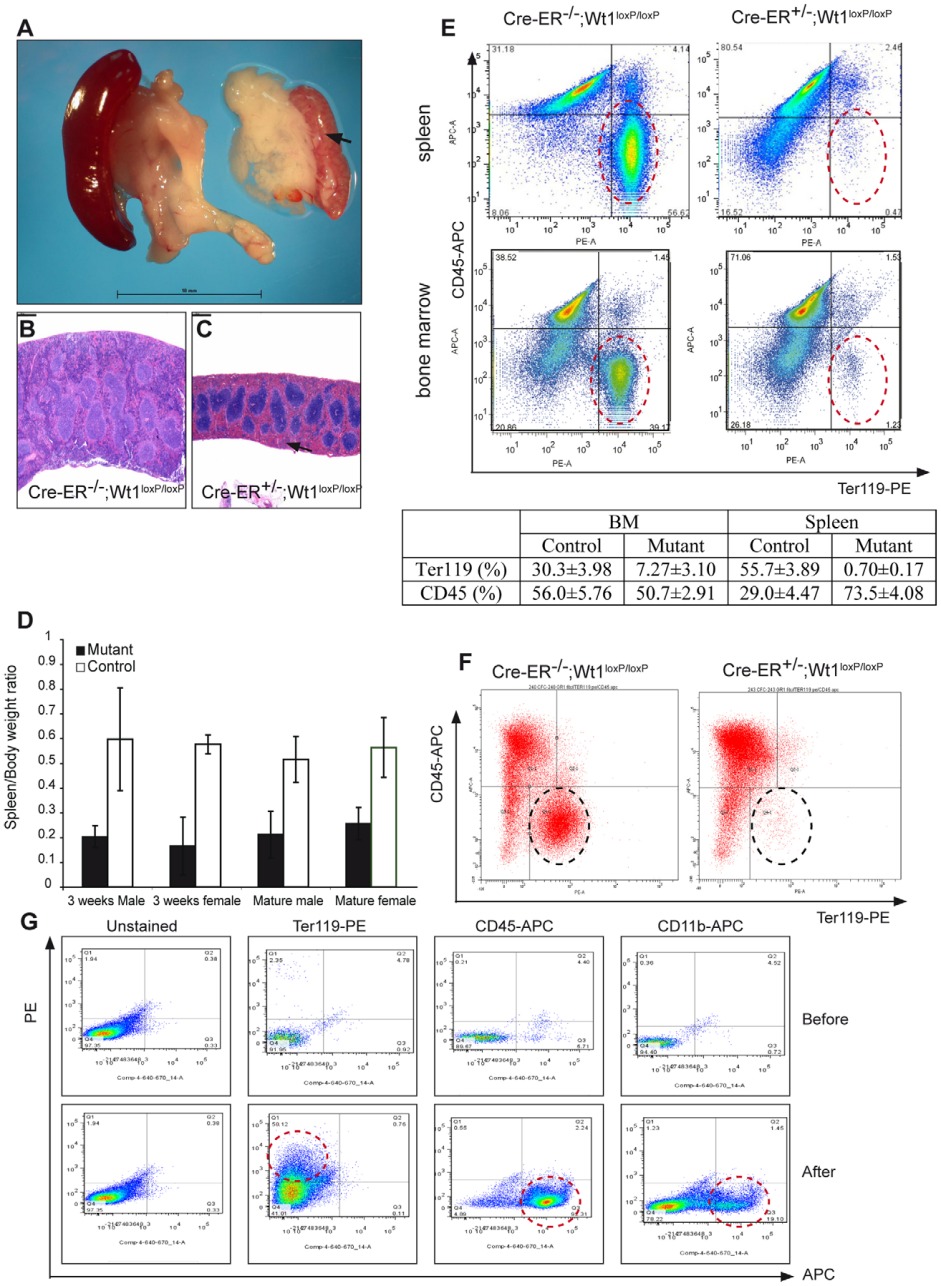
Deletion of *Wt1* leads to an aberrant haematopoietic system. A, Images of mutant spleen (arrow) compared with control spleen (injected at 3 week old); scale bar, 10 mm. (B,C) H&E staining show depletion of the red pulp compartment in the mutant spleen (C, arrow) c.f. in the control spleen (B; injected at 10 week old);scale bar, 400 µm. D, Analysis of the spleen to body weight ratio (control, black; mutant, white box). E, FACS analysis of spleen and bone marrow cells with CD-45 APC(y-axis) and Ter-119 PE(x-axis, red circles). The percentage of CD45 and Ter119 positive cells is summarised in the table. F, FACS analysis of Ter119-PE and CD45-APC on control and mutant bone marrow cells grown in a methylcelluose based medium, where Wt1 is deleted *in vitro* by culturing with 4-OH tamoxifen (1 µM). G, FACS analysis of Ter119, CD45, and CD11b on FACS-sorted GFP positive bone marrow cells (from *Wt1*-GFP knockin mice) before and after grown in a methylcellulose-based system.

Asplenia in the conventional *Wt1*-null mice correlates with enhanced apoptosis in the primordial spleen cells [29]. In the adult *Wt1* KO model, the mutant spleen was much paler and smaller in size compared with the control spleen (Figure 2A, arrow). There was a reduction in the number of proliferating cells in the mutant spleen; however the number of cells expressing an apoptotic marker (active caspase 3) remained unchanged (Figure S8A–S8D). The spleen to body weight ratio was reduced by 60% in the mutants of both the young (Figure 2D, 3 week old, *p*-value = 0.003; 8 controls and 5 mutants were analysed) and mature groups (Figure 2D, *p*-value = 0.000, 9 controls and 12 mutants were analysed).

The mutant mice had diminished extramedullary haematopoiesis within the red pulp compartment while white pulp remained largely unaffected (Figure 2B, 2C). FACS analysis showed an almost complete absence of erythrocytes (Ter-119 positive) in the mutant spleens (Figure 2E, 0.69±0.17% in the mutant c.f. 55.7±3.9% in the control spleen, *p*-value = 0.024; five controls and three mutants were analysed) and in *Wt1*-mutant bone marrow (Figure 2E, 7.3±3.1% in the mutant c.f. 30.3±4.0% in the control bone marrow, *p*-value = 0.025; five controls and three mutants were analysed).

### An intrinsic defect in the mutant haematopoietic system

Maturation of red blood cells requires erythropoietin (EPO) [30], which is synthesised mainly in the kidney. Furthermore, Wt1 has been shown to transcriptionally activate the *EPO* gene [31]. To determine whether the defect in erythropoiesis is intrinsic to the haematopoietic system, we cultured the mutant bone marrow cells in a methylcellulose-based system where a complete set of factors for supporting haematopoietic differentiation is provided in the medium. After two weeks in culture, despite the presence of all the required growth factors, the *Wt1*-mutant bone marrow cells failed to differentiate into the erythrocyte lineage, while the control bone marrow cells, as expected, did form red blood cells (Figure 2F, 5.0%±1.87% in the mutant compared with 31.3%±9.6% in the control; five controls and three mutants were analysed, *p*-value = 0.05).

To address whether this defect in erythropoiesis reflects a cell autonomous role for Wt1 in haematopoiesis, we set out to characterise the 1% of bone marrow cells that express Wt1. Using the *Wt1*-GFP knockin mouse (*Wt1* ^GFP/+^), we FACS sorted GFP positive cells from the bone marrow of *Wt1* ^GFP/+^ mice and cultured them in a methylcellulose-based system. It has been shown previously that some Wt1-expressing cells in the bone marrow express markers characteristic of short-term haematopoietic stem cells (Ter119^−^CD45^+^Mac-1^lo^c-kit^+^Sca-1^+^) [18] but the differentiation potential of these cells was not investigated. Hence we investigated the potential of these Wt1-GFP cells to differentiate to different haematopoietic lineages in culture. First we stained the GFP positive BM cells with a set of haematopoietic stem cell markers (CD150, CD48, and CD244) [32] and showed that approximately 50% of GFP positive BM cells were in the population of oligolineage-restricted progenitors (CD150^−^CD48^+^CD244^−^). Before culturing, no GFP-positive cells were positive for Ter-119 or Cd11b and only a few percent of the cells expressed CD45. After two weeks in culture, the GFP-positive cells were able to form Ter119 (red blood cells), CD45 (white blood cells), and CD11b (granulocytes) positive cells (Figure 2G). From this we can conclude that the Wt1-expressing cells are oligolineage-restricted progenitors.

**Figure 3:**
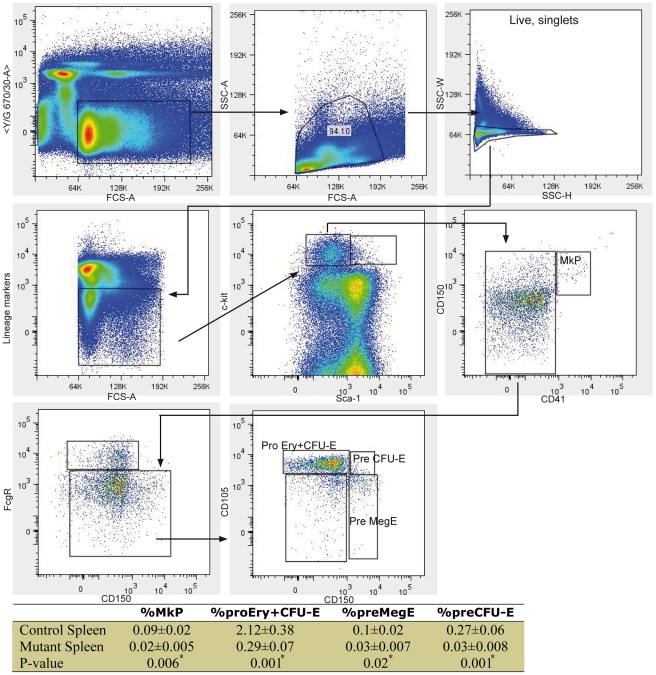
High-resolution fractionation of erythroid progenitors in mutant spleen. Spleen cells were stained with antibodies against Sca-1, c-kit, CD41, CD150, FcgR, CD105, and a cocktail mixture of mature blood cell lineage markers (Lineage). Cells were also stained with 7-AAD and only live cells are displayed. Representative flow cytometric profiles are illustrated. The percentage of MkP, Pro Ery+CFU-E, Pre MegE, and Pre CFU-E in control and mutant spleens is listed.

We then set out to test if the reduction of erythrocytes reflected a decrease in the number of erythrocyte progenitors (Pre CFU-E) using the high resolution myeloerythroid progenitor cell staging method described by Pronk *et al* [33]. Representative flow cytometric profiles are shown in Figure 3. We saw a significant reduction in the % of Pre CFU-E in the mutant spleen (Figure 3, 0.27±0.06 in the controls and 0.03±0.008 in the mutants, *p*-value = 0.001; 7 control and 8 mutant mice were analysed). Erythrocyte progenitor cells branch from megakaryocyte-erythrocyte progenitors (PreMegE). Another progenitor that branches from PreMegE is the Megakaryocyte progenitor (MkP) which produces platelets. Both MkP and Pre MegE were reduced significantly in the mutant spleen (Figure 3) However, the number of platelets in the circulation was not affected (control platelet number is 880.5±89.9 K/µL and mutant platelet number is 817.5±164 K/µL). Mutant mice did not show any obvious bleeding tendencies. The half life of platelets is about 35 hours [34]. Platelet deficiency may have developed if the mice had survived longer.

### Deletion of *Wt1* leads to rapid bone loss

**Figure 4:**
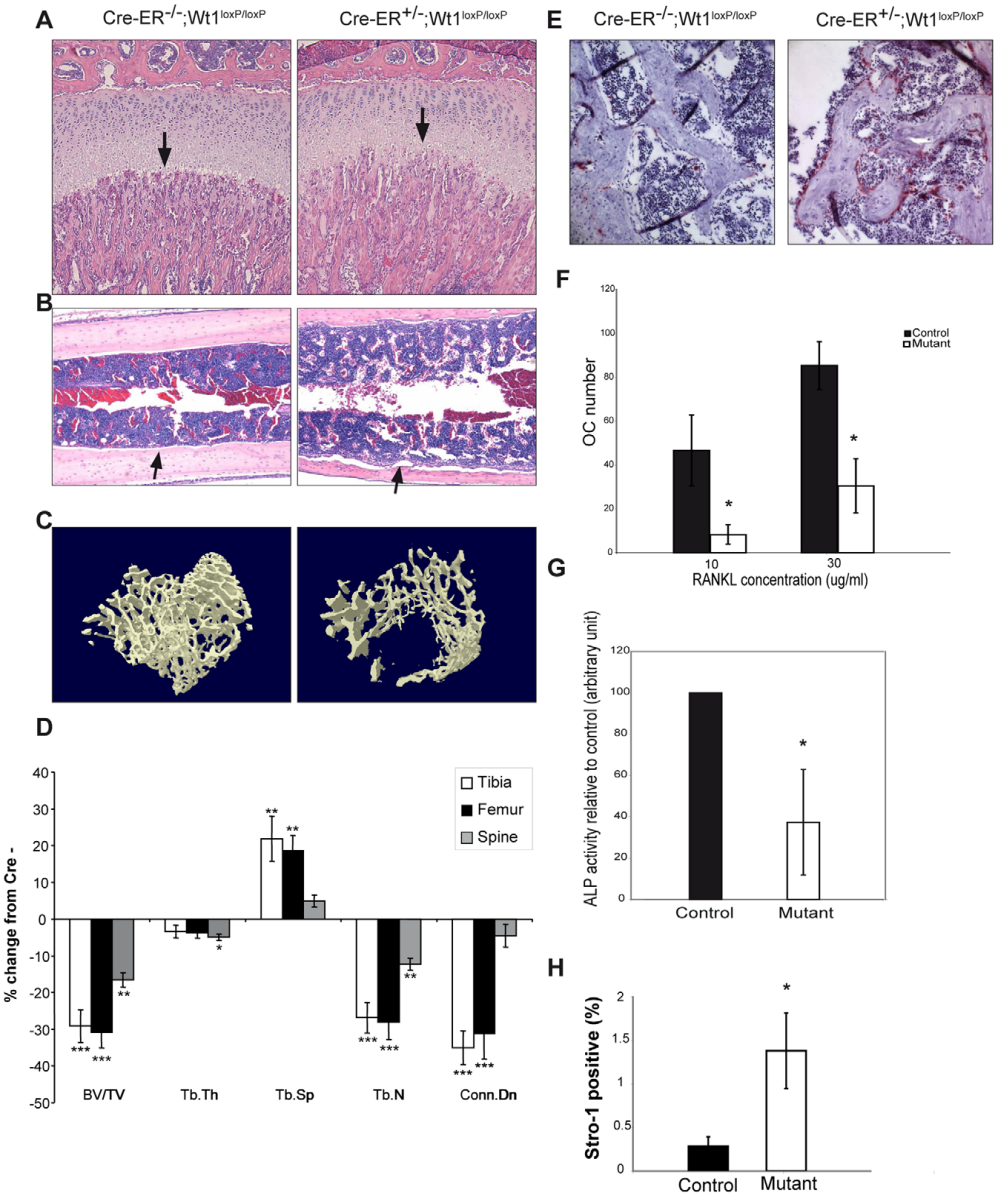
Deletion of *Wt1* leads to rapid bone loss. A, H&E staining show defects in the *Wt1*-mutant growth plates (arrows; injected at 3 week old). B, H&E sections of long bone from control and mutant mice (injected at 3 week old). C, uCT images of trabecular bone of femurs from mutant (right) and control mice (left) injected at 10 weeks old. D, Bone histomorphometry analysis on tibia, femur, and spine. Values are expressed as % of change from control mice (8 mutants and 8 control mice were analysed). BV/TV: percentage trabecular bone volume; Tb.Th: Trabecular thickness; Tb.Sp: Trabecular spacing; Tb.N: Trabecular number; Conn.Dn: Connectivity density. *:p<0.05; **:p<0.01; ***:p<0.001. E, TRAcP staining (red) showing osteoclasts in the bone section. F, Analysis of *in vitro* osteoclast formation ability from control and mutant bone marrow cells in the presence of RANKL at various concentrations (10 and 30 µg/ml). G, Analysis of alkaline phosphatase activity in osteoblasts, differentiated from bone marrow cells. H, FACS analysis of % of Stro-1 positive cells in control and mutant bone marrow.

We observed abnormalities of the growth plate in both the tibias and femurs of *Wt1*-mutant mice. The vascular invasion zones were irregular and anaemic (Figure 4A, indicated by arrow). The proliferative zone chondrocytes of the mutant mice were irregular with less surrounding territorial matrix than control mice (Figure 4A). The inner (marrow) surface of the long bone from the mutant mice was ragged compared with control mice (Figure 4B, arrows), suggesting increased bone resorption. We then analysed the bone architecture of femurs, tibias, and spine 9 days after induction of Wt1 deletion using μCT (Figure 4C). The 3D movie of the trabecular bone loss is shown in Videos S1 and S2. Trabecular bone volume was reduced by 30% in the mutants (Figure 4D), mostly due to a reduction in trabecular number and a small reduction in trabecular thickness. Furthermore, trabecular connectivity was also reduced. Taken together, these changes in bone architecture would be expected to lead to a substantial reduction in bone strength (Figure 4D). The bone loss observed could be due to either reduced bone growth or increased bone absorption. However, bone formation is a relatively slow process, and in view of the rapidity of the phenotype observed here it seemed that increased bone resorption was the more likely cause. We therefore stained sections of the long bones for the osteoclast marker TRAcP and observed dramatically increased numbers of osteoclasts on the bone surface of the *Wt1*-mutant bones (Figure 4E). To test if these bone phenotypes might reflect an intrinsic role for Wt1 in the osteoclast and osteoblast lineages, we harvested fresh bone marrow cells from the mutant mice, induced Wt1 deletion by treating the bone marrow cells with 4-OH tamoxifen for three days and cultured the cells in media supplemented with M-CSF and RANKL to induce osteoclast differentiation. Surprisingly and in contrast to the *in vivo* study, mutant bone marrow cells in which Wt1 had been deleted by tamoxifen treatment were less capable of forming osteoclasts *in vitro* (Figure 4F, *p*-value = 0.05 and 0.029 at 10 and 30 µg/ml of RANKL respectively; three separate experiments were performed each using bone marrow pooled from 2–3 control or mutant mice). When we used a similar *in vitro* approach using culture medium inducing osteoblastic differentiation, we observed that *Wt1*-mutant osteoblasts had reduced bone differentiation ability as levels of the osteoblast marker enzyme alkaline phosphatase were reduced (Figure 4G, *p*-value = 0.037; three separate experiments were performed). These results suggest that Wt1 plays an intrinsic role in both osteoclast and osteoblast differentiation, and that the loss of Wt1 is likely to disturb bone homeostasis.

### Fat reduction following Wt1 deletion

**Figure 5:**
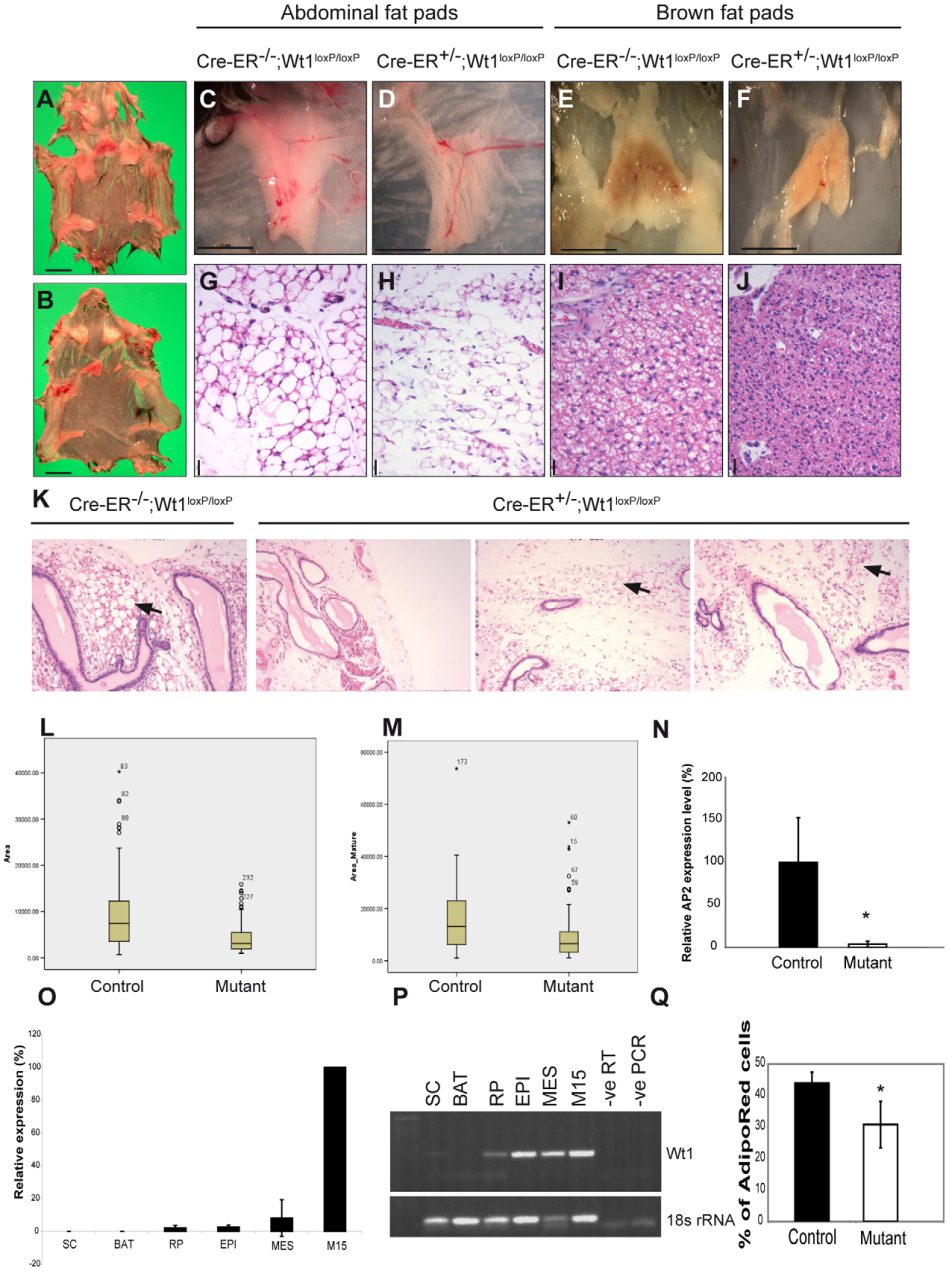
Fat reduction following Wt1 deletion. Skin pulps from control (A) and mutant (B) mice (injected at 3 week old); scale bar, 1 cm. (C,D) Images of abdominal fat pads. (E,F) Images of interscapular brown adipose tissue; scale bar, 5 mm. H&E staining of the corresponding fat pads is shown in G–J, respectively; scale bar, 25 µm. K, H&E sections of abdominal fat pads from mice injected at 13 week old (arrows indicate lipid vacuoles). Box plot of lipid vacuole size measurement of adipocytes in the abdominal fat pads from the younger group of mice (L) and from the matured group of mice (M). N, Quantitative PCR analysis of AP2 expression in the abdominal fat pads in control and mutant mice. O, Quantitative PCR analysis of relative level of Wt1 expression in different fat pads. P, RT-PCR showing Wt1 and 18s rRNA expression in fat pads. SC, subcutaneous; BAT, brown adipose tissue (interscapular brown adipose tissue); RP, retroperitoneal; EPI, epididymal; MES, mesenteric; M15, murine embryonic mesonephros-derived cell line (positive control for Wt1 expression). Q, FACS analysis of number of adipocytes positive for AdipoRed in control and mutant bone marrow (*p*-value = 0.018).

The *Wt1*-mutant mice also displayed reduction in the size of fat pads. In addition to the abdominal fat pads which mainly comprise white adipocytes, interscapular brown adipocytes were also atrophic and had fewer lipid cytoplasmic vacuoles than controls (Figure 5A–5J). Although the trend of fat loss was consistent in mutant mice, the reduction of fat pad size seemed to be more variable in the older group of animals (13 weeks, Figure 5K, arrows). In some mutant animals, the reduction in the size of fat pads was observed in both the interscapular and abdominal fat pads, while in other mutants the lipid vacuole size reduction was seen in the abdominal fat pads but not in the interscapular fat pads. The weight of fat pads in the mutant mice did not reflect their actual size because of the oedema (data not shown), and we therefore analysed fat pad volume using whole body μCT scans. Mice were scanned at the start (before tamoxifen injection) and the end of the experiment (9 days after induction). Results from the μCT scan confirmed the substantial fat loss in the mutants (Figure S7, arrows). There was no difference in the number of apoptotic and proliferating cells in the fat pads between mutant and control mice. Histological analysis of the adipose tissues showed that the reduction in the size of fat pads reflected a decrease in the vacuole size of the adipose tissues, as seen in the abdominal fat pads (Figure 5L–5M, p<0.05; three controls and three mutant mice were analysed). Consistent with this loss of fat, there was a significant reduction in the level of AP2 expression in mutant abdominal fat pads (Figure 5N, *p*-value = 0.05; three controls and three mutants were analysed).

Wt1 expression in fat has not been reported previously. However, here we show that Wt1 is expressed in the mesentery, epididymal, and retroperitoneal fat pads, but not at detectable levels in the abdominal fat pad nor in the interscapular brown adipose tissue (Figure 5O, 5P). Given the fact that adipocytes and osteoblasts have a common origin in the bone marrow, we examined whether there was any alteration in the number of adipocytes in the bone marrow. Labelling adipocytes using AdipoRed, we found a reduction in the number of adipocytes in the mutant bone marrow (Figure 5Q, *p*-value = 0.021; four controls and four mutants were analysed).

As adipocytes and osteoblasts arise from the stromal mesenchymal population in the bone marrow, we speculated that Wt1 loss might lead to a disturbance in this population which can be quantified using an antibody to Stro-1. We did in fact find a significant (five fold) increase in this population of cells following Wt1 loss (Figure 4H, *p*-value = 0.02; four controls and four mutants were analysed).

### Deletion of *Wt1* leads to atrophy in the exocrine pancreas

**Figure 6:**
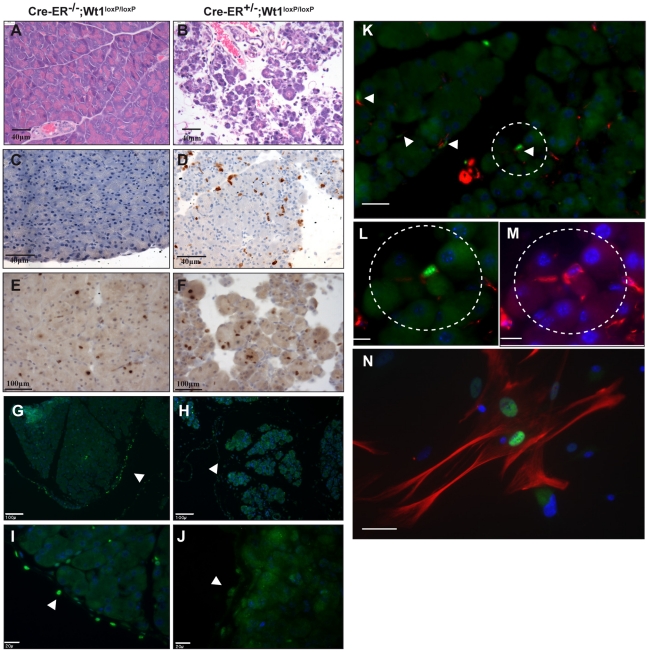
Deletion of *Wt1* leads to atrophy in the exocrine pancreas. (A, B) H&E staining show massive atrophy in the exocrine pancreas. Immunohistochemistry analysis show active caspase 3 (C,D; scale bar, 40 µm); Ki67 (E,F; scale bar, 100 µm) and Wt1-antibody (G,H). Nuclei are stained with DAPI (blue); scale bar, 100 µm. Higher magnification images are shown in I&J scale bar, 20 µm. K, Double immunofluorescence staining of pancreas sections with Wt1-antibody (green)and desmin antibody (red). Nuclei are stained with DAPI (blue); scale bar, 25 µm. Area circled in (K) is shown in a higher magnification in L&M; scale bar,10 µm. N, Double immunofluorescence of Wt1(green) and desmin (red) in cultured PSCs; scale bar 50 µm.

Figure 6G–6J (arrows) shows the successful depletion of Wt1 expression in the pancreatic mesothelium. The pancreas from the mutant mice was severely atrophied. H&E staining demonstrated that there was a substantial amount of cell loss in the exocrine tissues while the endocrine pancreas remained largely unaffected (Figure 6A, 6B). Acini in the mutant pancreas were loosely packed and acinar cells appeared atrophied and presented less eosinophilic cytoplasmic staining, suggesting a reduced zymogen content. Residual acinar epithelial cells were rounded and less cohesive with neighbouring cells. Similar aberrant histology started to appear at day 7 after Cre activation (Figure S4B). We saw an increase in the number of apoptotic cells in the mutant pancreas (Figure 6C, 6D) while the number of proliferating cells remained unchanged (Figure 6E, 6F). Although the pathology of our model shares many similarities to pancreatitis mouse models, there was no elevation of serum amylase level in the *Wt1*-mutant mice (Table 2). Given the severity of the pancreas phenotype, it is surprising to see the lack of any elevation of serum amylase. However, this probably reflects the short space of time between the onset of the phenotype and death of the mice. Pancreatitis involves inflammation of the pancreatic tissues and in *Wt1*-mutant mice we observed a low-grade inflammation in much of the pancreas and scattered foci of more severe active inflammation. In the *Wt1*-mutant pancreas, the presence of infiltrating macrophages was confirmed by staining with macrophage marker F4/80 (Figure S6E, S6F); however, staining of CD11b, Gr1, and CD3 were absent (data not shown). Both insulin and amylase expression were normal in the mutant pancreas sections (Figure S6A–S6D).

To try to gain more insight into the origin of the pancreatic phenotype, we examined more closely the cell types that express Wt1 in the exocrine pancreas. Pancreatic stellate cells (PSCs) have been implicated in pancreatitis and pancreatic cancer. We show Wt1 is expressed in the mesothelial lining of the pancreas as well as in PSCs. Desmin is a marker for PSCs [35]. The interstitial cells that express Wt1 also express desmin, and this was demonstrated in sectioned pancreata (Figure 6K–6M) and in cultured PSCs (Figure 6N).

### Serum protein profiling reveals no systemic inflammatory or nutritional response but dramatic reduction in IGF-1 levels

**Figure 7:**
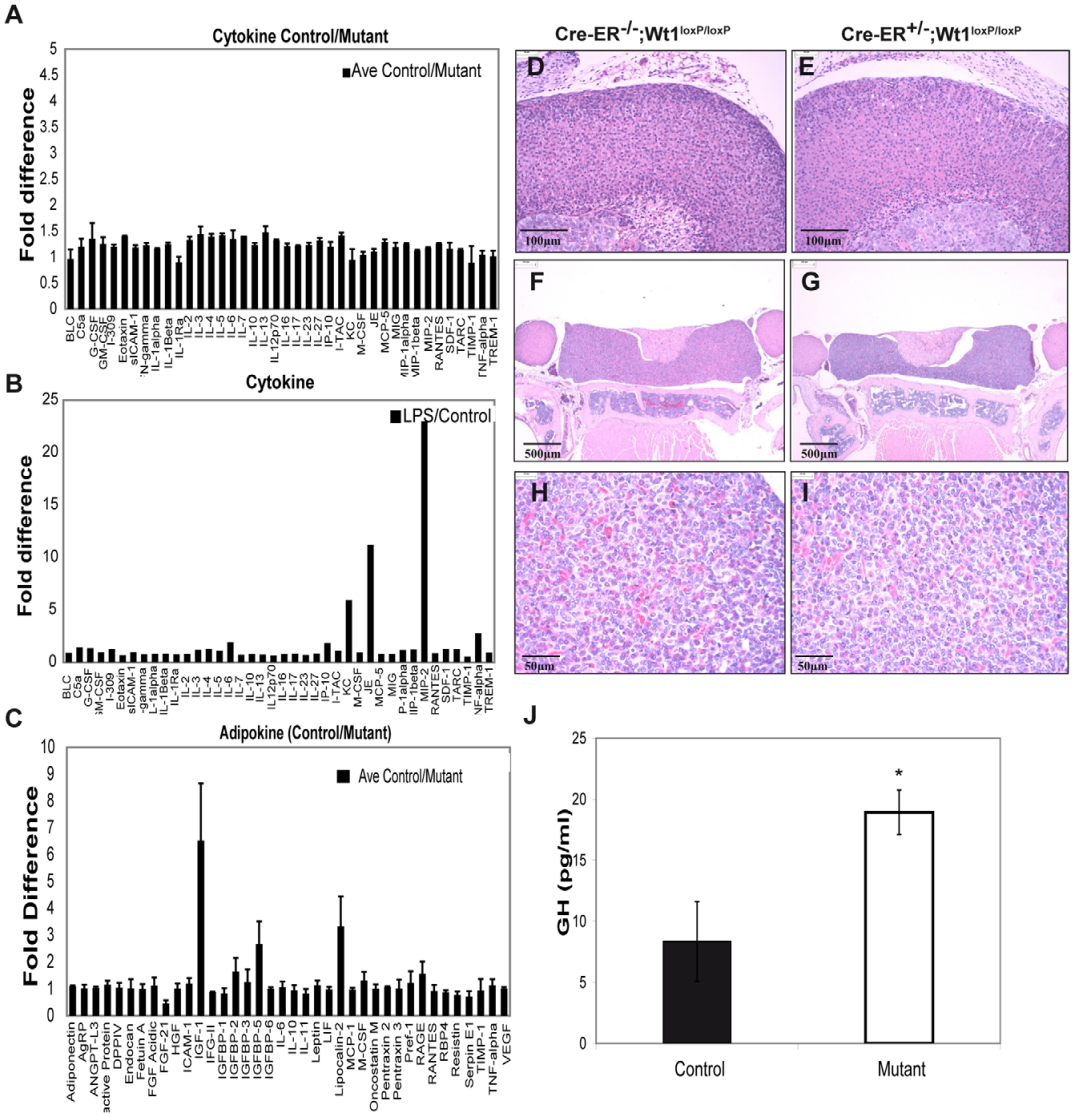
Cytokine, adipokine, and growth hormone analysis in control and mutant plasma. A, Cytokine profiling in control and mutant plasma. B, Cytokine array performed using plasma from mouse treated with LPS. C, Results from adipokine array showing fold of difference in the level of adipokines between mutant and control mouse plasma. D &E, H&E staining of adrenal glands (control = left, mutant = right; scale bar = 100 µm). F&G, H&E staining of pituitary glands; scale bar = 500 µm. H&I, H&E staining of pituitary anterior lobes; scale bar = 50 µm. J, Measurement of growth hormone (GH) in mouse plasma using ELISA.

One possible explanation for the dramatic and acute nature of the phenotypes observed in these mice is a systemic inflammatory response, even though analysis of the diseased pancreas did not suggest this. Furthermore, even though the animals appeared to show no signs of distress and their stomachs were full at 9–10 days, it is possible that the bone and fat defects were due to nutritional deprivation. To assess these possibilities, we carried out quantitative analysis of 40 cytokines and 38 adipokines in mutant versus wildtype serum using antibody arrays. Perhaps surprisingly, given the severity of the phenotypes there was no statistically significant change in the levels of any inflammatory cytokines (Figure 7A; three controls and three mutants were analysed), arguing that the phenotypes were not due to a systemic inflammatory response. As a positive control to test that the arrays were working, we treated the mice with LPS and then assayed cytokine levels. There was a 23 fold induction in MIP-2, an 11 fold induction of JE, a 6 fold induction of KC, and a 3 fold induction of TNFα (Figure 7B). These findings demonstrate that the assays work and are able to measure an acute systemic inflammatory response. Similarly, there was no indication of nutritional deprivation. Following calorific restriction, there is reported to be a 60–80% reduction in serum leptin levels [36], [37], a 65% reduction in TNFα [38], a 100% increase in AgRP/FIAF [39], and a 75% increase in the levels of adiponectin [40]. We saw no significant changes in any of these molecules (Table 3 and Figure 7C), supporting the idea that the mice were not suffering nutritional deprivation and, in turn, this was not causing any of the phenotypes. However, we did observe a dramatic 85% reduction in the levels of IGF-1 and 3.5 fold increase in the levels of FGF21 (Figure 7C). This could in part account for the bone and fat phenotypes respectively. To investigate if the reduction of IGF-1 levels could due to global growth hormone deficiency, we measured circulating growth hormone (GH) using ELISA. We observed a slight elevation of GH levels in the mutant serum (Figure 7J; three controls and five mutants were analysed, *p*-value = 0.025). Histology analysis showed absence of any pathological abnormalities in the pituitary and adrenal glands (Figure 7D–7I).

**Table 3:** Comparison of change of adipokine levels in fasting/caloric restriction condition and adult mice deleted for *Wt1*.

	Fasting/Caloric restriction	Wt1 deletion
Leptin	60–80% reduction [36], [37]	No change
Adiponectin	75% increase [40]	No change
TNF-α	65% reduction [38]	No change
AgRP/FIAF	100% increase [39]	No change
IGF-1	30% reduction [56], [57]	85% reduction

## Discussion

The multiple organ disturbance observed in adult mice deleted for Wt1 is striking, and, we believe, unprecedented in terms of severity and rapidity of onset. There is perhaps no need to point out that most of these phenotypes have relevance for diseases common in adults, even though our starting point was a gene more or less defined for its role in the development of several organs. Our study shows that Wt1 plays a key role in regulating the production or turnover of red blood cells, bone and fat in the adult. Despite intensive analysis of *Wt1*-null foetuses, including those surviving to 18 days gestation, no developmental defects in these tissues were found previously [4], [29]. Thus our study contributes to the growing body of evidence that adult tissues may employ different or additional players compared to foetal development. Wt1 is among a list of genes whose methylation increases with age in a genome-wide CpG island methylation profiling study [41]. Therefore Wt1 expression levels may well decrease with age. It will be important to determine whether Wt1 levels in these key cell populations reduce during aging or under different environmental influences. If so, this could contribute to disease-related phenotypes described here.

Although there is much future work needed to elucidate the mechanisms underlying these phenotypes, there are several conclusions we can draw at present. Perhaps, surprisingly, we could detect no significant changes in serum cytokine levels, arguing that the phenotypes we observe are unlikely to be due to a systemic inflammatory response, even though this is often associated with damage to the tissues that are affected in the Wt1 mutant mice. As we argue below, the phenotypes involving the kidney and erythrocytes reflect an intrinsic function of Wt1 in these tissues or their progenitors. On the other hand, we believe loss of fat and bone is likely to be a combination of systemic and local factors.

The phenotypes involving the haematopoietic system and bone, have their origins wholly or partly within the bone marrow itself. Wt1 is expressed in a restricted haematopoietic progenitor population and its loss leads to disturbance in red blood cell and osteoclast production. This is consistent with the previous finding that Wt1 expression is upregulated during early myeloid differentiation (particularly in the common myeloid progenitors and megakaryocyte-erythroid progenitors) [18]. In keeping with this, we found the levels of PreMegE, MkP, and Pre CFU-E were significantly decreased in mutant spleen, Given the association of Wt1 with AML, we might have expected an imbalance in the myeloid compartment. Preliminary analysis has not demonstrated a reduction in the absolute number of monocytes and granulocytes in the circulation of Wt1 mutant mice. However, this may have only become evident if the mice had survived longer.

The bone loss in most part is likely to result from the increase in osteoclasts that we observed in the bone marrow. Paradoxically, mutant mice showed a reduction in osteoclast formation ability *in vitro*. The bone marrow compartment in which we saw an increase in the number of osteoclasts consists of a mixed population of cells. The mesenchymal stromal cells and haemaatopoietic stem cells are in close proximity in the bone marrow and there is known to be crosstalk between these cell types [42], [43]. Our *in vitro* osteoclast formation cell culture system started with a restricted population of cells (bone marrow stromal cells). The *in vivo* and *in vitro* difference could be due to factor(s) that are present in the bone marrow but absent in the *in vitro* culturing system.

However, we also found that Wt1 is required for osteoblast synthesis in bone marrow culture pointing to a role in the mesenchymal lineage. Consistent with this, our preliminary experiments have shown that non-haematopoietic Wt1-GFP positive cells from the bone marrow stroma are able to differentiate to bone and fat (unpublished observations). Furthermore, we show here that Wt1 loss also leads to an increase in Stro1 positive stromal mesenchymal stem cells, which may explain partly the disturbance in adipocyte and osteoblast production in the bone marrow. Our serum protein analysis showed a dramatic reduction of IGF-1 levels and this might be expected to contribute to the bone loss phenotype. Interestingly, deletion of IGF-1 specifically in the liver, the major source of synthesis, only leads to a 75% reduction in circulating IGF-1 levels and there is no apparent phenotype [44]. However, mice that are double homozygous mutant for IGF-1 and the binding protein acid labile subunit (ALS) [45] show an 85% reduction in IGF1- levels and a similar degree of bone thinning to that seen in our Wt1 adult knockout mice. Hence, it seems reasonable to conclude that the 85% reduction of IGF-1 levels in our mutant mice is a major factor behind the bone phenotype. In the Wt1 mutant mice, the IGF-1 levels are much lower than those observed when IGF-1 is deleted specifically in the liver, so either Wt1 is required for IGF-1 expression in non-hepatocytes, or for factors that stabilise IGF-1 in the serum. Growth hormone, produced by the pituitary gland, is a major regulator of IGF-1 levels. One possibility was that the reduction in IGF-1 level was due to defects in the pituitary axis and downregulation of GH. However, we detected no pathological abnormalities in the pituitary and adrenal glands, and if anything GH levels were increased.

Obesity is a major health problem and there is considerable topical interest in the factors that regulate fat levels. Loss of Wt1 not only leads to reduced adipocyte production in the bone marrow but also to rapid systemic loss of fat, with dramatically reduced vacuole size. There are several reasons why we believe this fat loss is not due to under-nourishment. Fat vacuole reduction was already apparent 7 days after tamoxifen injection, at which time the health status of the animals was normal. Nine days post-injection, the mutant mice still actively sought food and their stomachs were full on autopsy. Importantly, there was no change in the levels of circulating leptin, adiponectin, TNFα, and AgRP/FIAF, all of which would be expected to change dramatically after one or two days of calorific restriction. There was a reduction in the level of lipocalin 2 in mutant serum (Figure 7C). Lipocalin 2 is abundantly produced from adipocytes [46], [47]. The reduction of lipocalin 2 could be caused by the reduced volume of adipose tissues in mutant mice. Taken together our findings provide evidence that Wt1 may influence both the formation and maintenance of adipocytes. The fat loss is extremely rapid and given that Wt1 only appears to be expressed in a proportion of fat pads affected, it seems likely that systemic factors might be involved. We found that the levels of circulating FGF21 increased by 3.5 fold in the mutant animals and this would be expected to induce some fat loss [48].

The RT-PCR result showed that Wt1 expression was detected in fat pads (Figure 5O). In preliminary experiments to address whether this reflects expression in mature adipocytes or the stromal vascular compartments, we digested and fractioned fat tissues from the Wt1-GFP knockin mice into the floating mature adipocyte layer and the stromal vascular fraction. The majority of the GFP signal was seen in the stromal vascular fraction (unpublished data). This supports the idea that systemic or local paracrine factors dependent on Wt1 are regulating adipocyte homeostasis.

The effect of Wt1 loss on bone and fat turnover is interesting in the context of Wilms' tumours. We and others have shown that the 15–20% subset of Wilms' tumours arising through WT1 loss are more likely to be stromal (mesenchymal) predominant and often contain ectopic tissues, including bone, fat, cartilage and muscle [49], [50], [51]. Taken together this and our new findings underline the key role of the mesenchyme and Wt1 in tissue turnover and maintenance.

With regard to the pancreatic atrophy, this does not appear to be typical pancreatitis as there was no increase in serum α-amylase. However, as discussed above, amylase level may have increased if the mice had lived longer. Serum cytokine profiles showed that there was no systemic inflammatory response in the mutant mice. In line with this there was no observable pathology in liver, lung and intestine, all tissues susceptible to inflammation. It remains to be seen whether the severe pancreatic atrophy is due to loss of Wt1 function within the tissue itself. We can exclude an effect through loss of Wt1 function in the islet or acinar cells as deletion of the gene specifically in these cell types using PDX1-Cre did not lead to overt pathology in the pancreas or elsewhere (P. Hohenstein, V. Brunton, M. Frame, O. Samson and N. Hastie unpublished observations). One possibility is that the pancreatic atrophy arises through activation of the sub-population of stellate cells that express Wt1 although further study is required to investigate this hypothesis. Activated stellate cells produce cytokines [52] and we speculate that these may be responsible for destroying the acinar cells. Given the published data on foetal liver [8], the parallel between pancreatic and hepatic stellate cells, and the role of Wt1 in generating vascular progenitors from the epicardium by EMT [5], we hypothesise that a proportion of pancreatic stellate cells arise from the mesothelium, via an EMT, once more pointing to the role of this tissue as a source of mesenchymal progenitor cells.

Despite the accumulating knowledge about the importance of Wt1 at multiple stages of kidney development, the function of Wt1 in the podocytes of mature glomeruli has remained the subject of some speculation. Even though children and adult mice with *Wt1* mutations characteristic of Denys-Drash and Frasier syndrome develop glomerulosclerosis, it was always possible that the damage had its origin in utero, rather than reflecting a continued function for Wt1 in the maintenance of the adult kidney. Our results provide the first evidence that Wt1 is crucial for maintaining the integrity of mature podocytes. Our model allowed us to test whether the glomerulosclerosis we observed arises through abnormalities of cell proliferation or the differentiation state of the mature podocytes. We did not see major changes in proliferation or apoptosis in the mutant glomeruli deleted for Wt1 (using proliferation marker anti-phosph-histone H3 and apoptosis marker active caspase 3, Figure S8E–S8H). However, we showed that loss of Wt1 expression resulted in damage to the foot processes of the podocytes therefore causing a morphological alteration. Nephrin is necessary for the renal filtration barrier and is also a known downstream target of Wt1 during kidney development [53]. Consistent with this we found that nephrin expression levels reduce dramatically after Wt1 deletion, indicating that the transcriptional regulation of nephrin by Wt1 continues into adult life. Here we show that Wt1, known to be a key regulator of nephrogenesis, is also vital for the maintenance of adult glomerular structure and function, something that has been the subject of speculation but not proven until now.

Clearly these findings should be followed up using tissue specific Cre lines. However, at present suitable Cre lines are not available for several of the crucial lineages we wished to investigate, including the mesothelium and mesenchymal stem cells. In the mean time, we have been able to use cultures to show that several of the phenotypes we observed are intrinsic to the bone marrow.

The results presented in this study open new avenues of research into mesenchymal cell function in adult tissues. The cell types that express Wt1 in adult tissues e.g. the hepatic and pancreatic stellate cells and bone marrow progenitors are mesenchymal. The other major cell types expressing Wt1, namely the podocytes and mesothelia are considered epithelial, but are unusual in expressing high levels of mesenchymal markers, such as vimentin. Given our findings, it is interesting to speculate on the possible relationships between the cell types expressing and requiring Wt1 in these different tissues. Different reports have shown that stellate cells may arise from the mesothelium and bone marrow [10], [54]. Our studies suggest that Wt1 may have a function in both stellate cells and bone marrow mesenchymal stem cells. Stellate cells, like the epicardially-derived cells requiring Wt1, synthesise retinoic acid. One of the striking features of stellate cells is the presence of vitamin A (retinoid) droplets and this becomes lost upon stellate cell activation. In the epicardium we have shown that RALDH2 levels and RA are reduced when *Wt1* is deleted and that RALDH2 is a direct transcriptional target of Wt1 [9]. We have shown that Wt1 is required for the EMT that generates RA-synthesising coronary vascular progenitors from the epicardium and it is interesting that an EMT is required for activation of stellate cells. It is also notable that stellate cells synthesise high levels of fat and it will be interesting to see if the Wt1 expressing cells in fat have similarities to stellate cells and mesenchymal bone marrow cells.

Finally our findings may also have implications for cancer therapy. There is a growing number of studies developing anti-WT1 immune therapy for common cancers predicated on the belief that WT1 is expressed at high levels in cancers [20], [55], but very low levels in the normal adult. Our findings raise questions about this approach as damage to these normal Wt1-expressing tissues might have adverse effects.

## Materials and Methods

### Generation of *Wt1*-conditional knockout mice

Mice were housed and bred in animal facilities at the MRC HGU and the University of Edinburgh. Animals were kept in compliance with Home Office regulations. The *Wt1*-conditional line was made in our group [5]. To obtain [*CAGG-CreER*™ positive, *Wt1* ^loxP/loxP^] and [*CAGG-CreER*™ negative, *Wt1* ^loxP/loxP^] transgenic mice, [*CAGG-CreER*™ positive] males were mated with *Wt1* ^loxP/loxP^ females, and the resulting offspring intercrossed. *Wt1*-GFP knockin mice used in this study were kindly provided by Professor H Sugiyama [18].

### Tamoxifen-induced *Wt1* deletion in [*CAGG-CreER*™, *Wt1* ^loxP/loxP^] mice

Cre recombinase was induced by intraperitoneal administration of tamoxifen (4 mg/40 g body weight for 5 days; Sigma). All animal work was carried out under the permission of license. To delete Wt1 *in vitro*, cells were treated with 4-OH tamoxifen (1 µM, Sigma) for three days.

Full methods are described in Text S1. Antibodies and primers are listed in Tables S1 and S2.

## Supporting Information

Figure S1 PCR testing for Cre-mediated recombination in inducible Wt1-KO. Top panel: PCR bands show the Cre-mediated recombination in all the tissues tested in the mutant mice (indicated by ‘recombination site’, CreER™^+/−^; Wt1^loxP/loxP^). The Cre-mediated recombination is not 100% as PCR bands represent no-recombination are still found in the mutant tissues (indicated by ‘lox site’). Lower panel: no recombination was detected in the control mice (tamoxifen injected litter mates, CreER™^−/−^; Wt1^loxP/loxP^).
(PDF)

Figure S2 Immunohistochemistry analysis of depletion of Wt1 expression in tissues from the mutant mice. Images of sections from mutant mice (CreER™^−/−^; Wt1^loxP/loxp^) are at the right column, and images from the control mice (litter mates, CreER™^−/−^; Wt1^loxP/loxP^) which have also been injected with tamoxifen are at the left column. Using a Wt1-specific antibody, Wt1 expression is detected in the mesothelial lining of organs including pancreas, spleen, lung, and uterus (brown, indicated by arrows). Wt1 expression is not detected in the corresponding tissues from the mutant mice. Scale bars, 20 µm in the heart, pancreas, and spleen. 100 µm in lung, and 40 µm in uterus.
(PDF)

Figure S3 Immunohistochemistry analysis indicate the intactness of the mesothelium in the mutant mice. Images of sections from the mutant mice are shown in the right column and images from the control mice are shown in the left column. Mesothelium lining of organs is detected using a cytokeratin antibody. Scale bars, 20 µm in the heart, pancreas, and spleen. Scale bar, 100 µm in the lung and kidney.
(PDF)

Figure S4 Characterisation of phenotypes in Wt1-KO mice at day 7 post-injection. H&E staining of sections from Wt1-KO mice. A, In the mutant kidney, protein casts are already visible. B, Moderate level of atrophy is seen in the mutant pancreas. C, The reduction in the size of fat vacuoles in the abdominal fat pad is already evident in the mutant mice. D, There is a slight reduction of the size of fat vacuoles in the brown fat pad from mutant mice; scale bars, 50 µm.
(PDF)

Figure S5 Minor gonadal defects in Wt1-KO mice. A, H&E staining show control (left) and mutant (right) testes. B, H&E staining of ovaries from control (left) and mutant mice (right). Follicles and corpora lutea are present in all mice but there was less luteal tissue in the mutant ovaries. In addition, there were fewer large, antral and atreic follicles in the mutant ovaries. Although the size of the gonads appear to be smaller in the mutants, the difference in the weights (e.g. testes) is not significant. Partial depletion of Wt1 expression in the Sertoli cells in the mutant testes (D) compared with the control (C). Wt1 staining in the granulosa cells in the ovaries (E) and its complete absence in the mutant (F). (G–I), Immunohistochemistry analysis of the expression of Sdmg1 (marker for Sertoli cells), Plzf1 (marker for spermatogonia), and Mvh (marker for spermatogonia, spermatocytes, and round spermatids) between control (left column) and mutant (right column) testes; scale bar, 50 µm.
(PDF)

Figure S6 Immunohistochemistry analysis of markers in the pancreas. Images of sections from the mutant mice are shown in the right column and images from the control mice are shown in the left column. A–D, Immunohistochemistry staining indicate normal insulin and α-amylase expression in the mutant pancreas; scale bar, 50 µm. E, F, Using a pan marker for macrophages (F4/80), infiltrating macrophages are detected in the mutant pancreas; scale bar, 40 µm.
(PDF)

Figure S7 Fat reduction in mutant Wt1-KO using μCT. Representative transverse images taken from the μCT scanned mice before and after tamoxifen injection. Control mice (CreER™^−/−^, Wt1^loxP/loxP^) used for fat analysis are the sexed matched littermates of the mutant mice (CreER™^+/−^, Wt1^loxP/loxP^). Light grey shades indicate fat tissues which are present in both control and mutant mice before tamoxifen injection (arrows). Darker shades indicate soft tissues and black shades indicate skeletons. Gaps indicate gastric gas trapped in the intestines of the animal. After 9 days of tamoxifen injection, a reduction in the fat pads is noticed in the mutant mice.
(PDF)

Figure S8 IHC staining of apotosis and proliferation markers in Wt1-KO mice. A,B, IHC staining of active caspase-3 in control (left) and mutant (right) spleen; scale bar = 50 um. C,D, IHC staining of phospho-histone H3 in control (left) and mutant spleen (right); scale bar = 100 um. E,F, IHC staining of active caspase-3 in control (left) and muatnt kidney (right); scale bar = 50 um. G,H, IHC staining of phospho-histone H3 in control (left) and mutant kidney (right); scale bar = 50 um. expression in the mutant pancreas; scale bar, 50 µm.
(PDF)

Table S1 Antibodies and dilution factor.
(PDF)

Table S2 Sequences of primers and Roche Universal Probe Library number used for Q-PCR.
(PDF)

Text S1 Supporting methods.
(DOC)

Video S1 Movie shows μCT scanned structures of trabecular bone of femurs from tamoxifen-injected control mouse.
(MOV)

Video S2 Movie shows μCT scanned structures of trabecular bone of femurs from tamoxifen-injected mutant mouse.
(MOV)
