# Supplementary figures and images for: Acute Multiple Organ Failure in Adult Mice Deleted for the Developmental Regulator Wt1
Source: PLoS Genet. 2011 Dec 22;7(12):e1002404. doi: 10.1371/journal.pgen.1002404 (PMC3245305; doi:10.1371/journal.pgen.1002404)

Figure S1

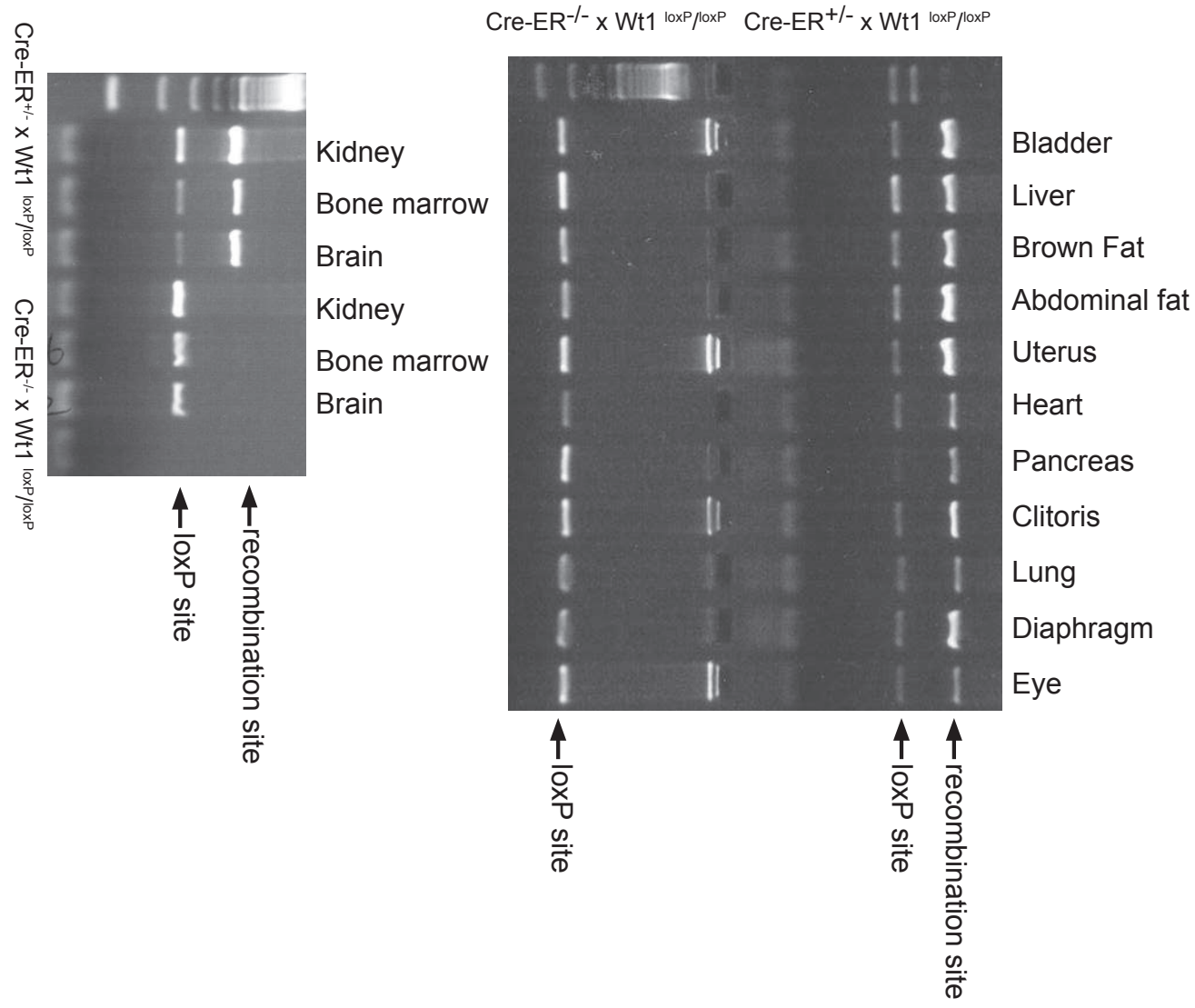

Supplement: Figure S1 — PCR testing for Cre-mediated recombination in inducible Wt1-KO. Top panel: PCR bands show the Cre-mediated recombination in all the tissues tested in the mutant mice (indicated by ‘recombination site’, CreER™+/−; Wt1loxP/loxP). The Cre-mediated recombination is not 100% as PCR bands represent no-recombination are still found in the mutant tissues (indicated by ‘lox site’). Lower panel: no recombination was detected in the control mice (tamoxifen injected litter mates, CreER™−/−; Wt1loxP/loxP). (PDF) [file pgen.1002404.s001.pdf]

Figure S2

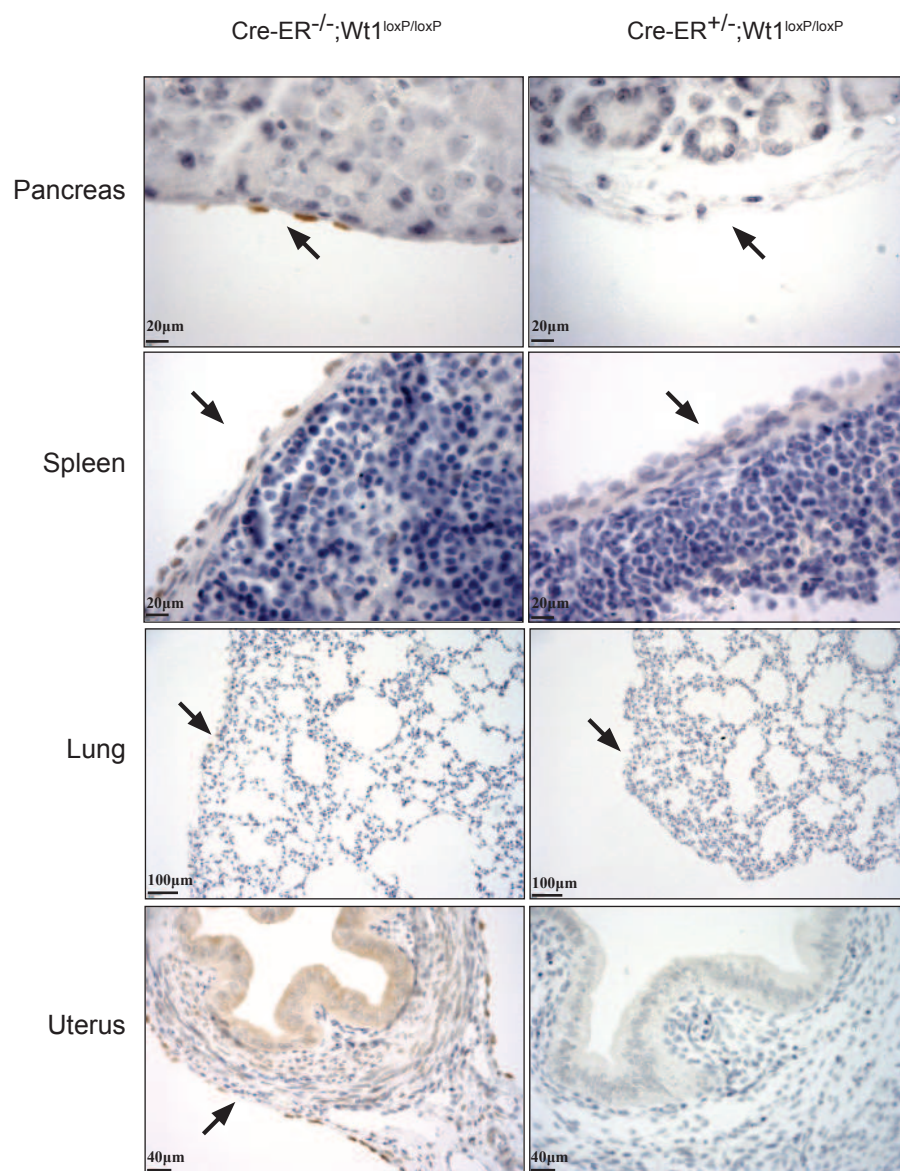

Supplement: Figure S2 — Immunohistochemistry analysis of depletion of Wt1 expression in tissues from the mutant mice. Images of sections from mutant mice (CreER™−/−; Wt1loxP/loxp) are at the right column, and images from the control mice (litter mates, CreER™−/−; Wt1loxP/loxP) which have also been injected with tamoxifen are at the left column. Using a Wt1-specific antibody, Wt1 expression is detected in the mesothelial lining of organs including pancreas, spleen, lung, and uterus (brown, indicated by arrows). Wt1 expression is not detected in the corresponding tissues from the mutant mice. Scale bars, 20 µm in the heart, pancreas, and spleen. 100 µm in lung, and 40 µm in uterus. (PDF) [file pgen.1002404.s002.pdf]

Figure S3

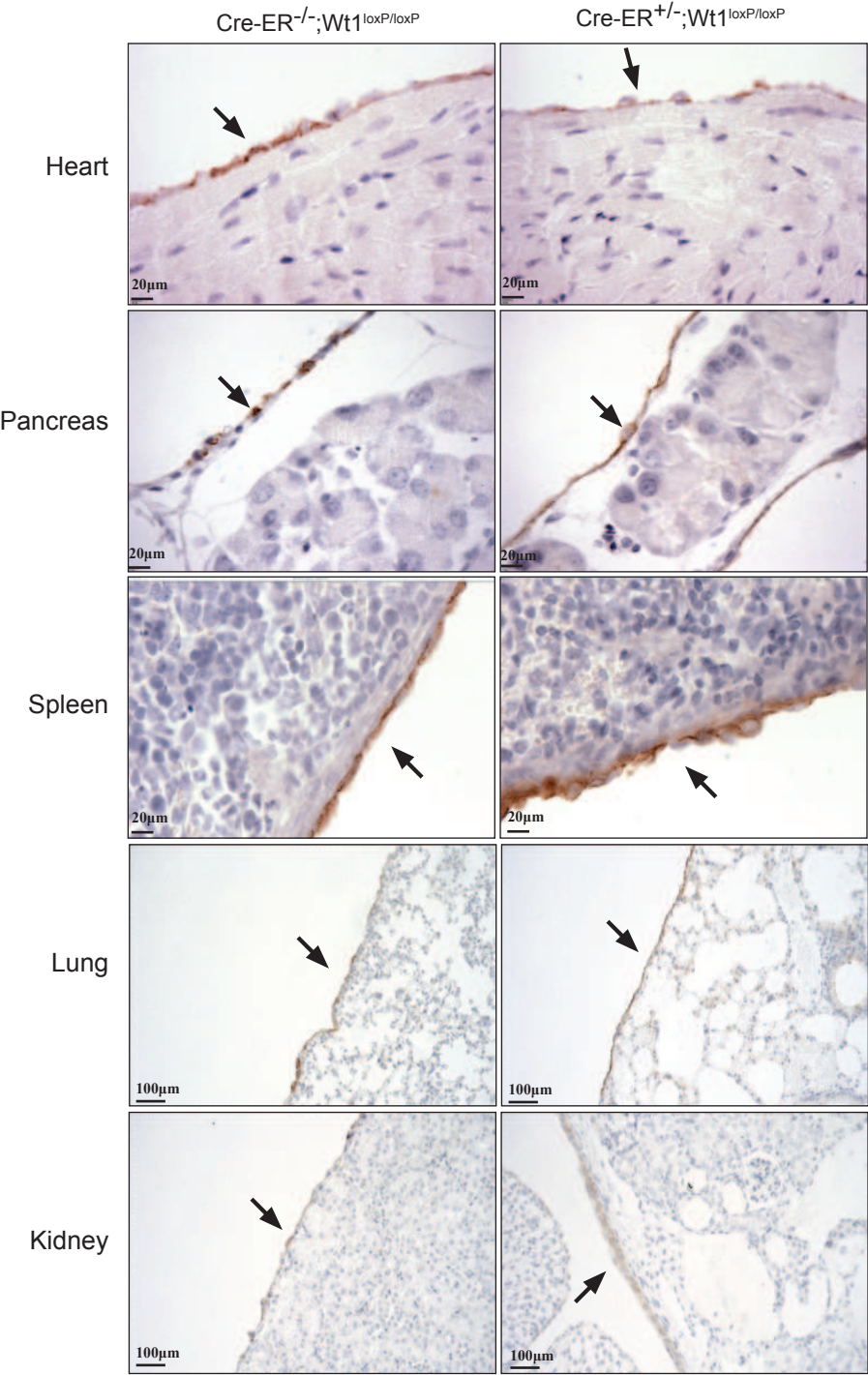

Supplement: Figure S3 — Immunohistochemistry analysis indicate the intactness of the mesothelium in the mutant mice. Images of sections from the mutant mice are shown in the right column and images from the control mice are shown in the left column. Mesothelium lining of organs is detected using a cytokeratin antibody. Scale bars, 20 µm in the heart, pancreas, and spleen. Scale bar, 100 µm in the lung and kidney. (PDF) [file pgen.1002404.s003.pdf]

Figure S4

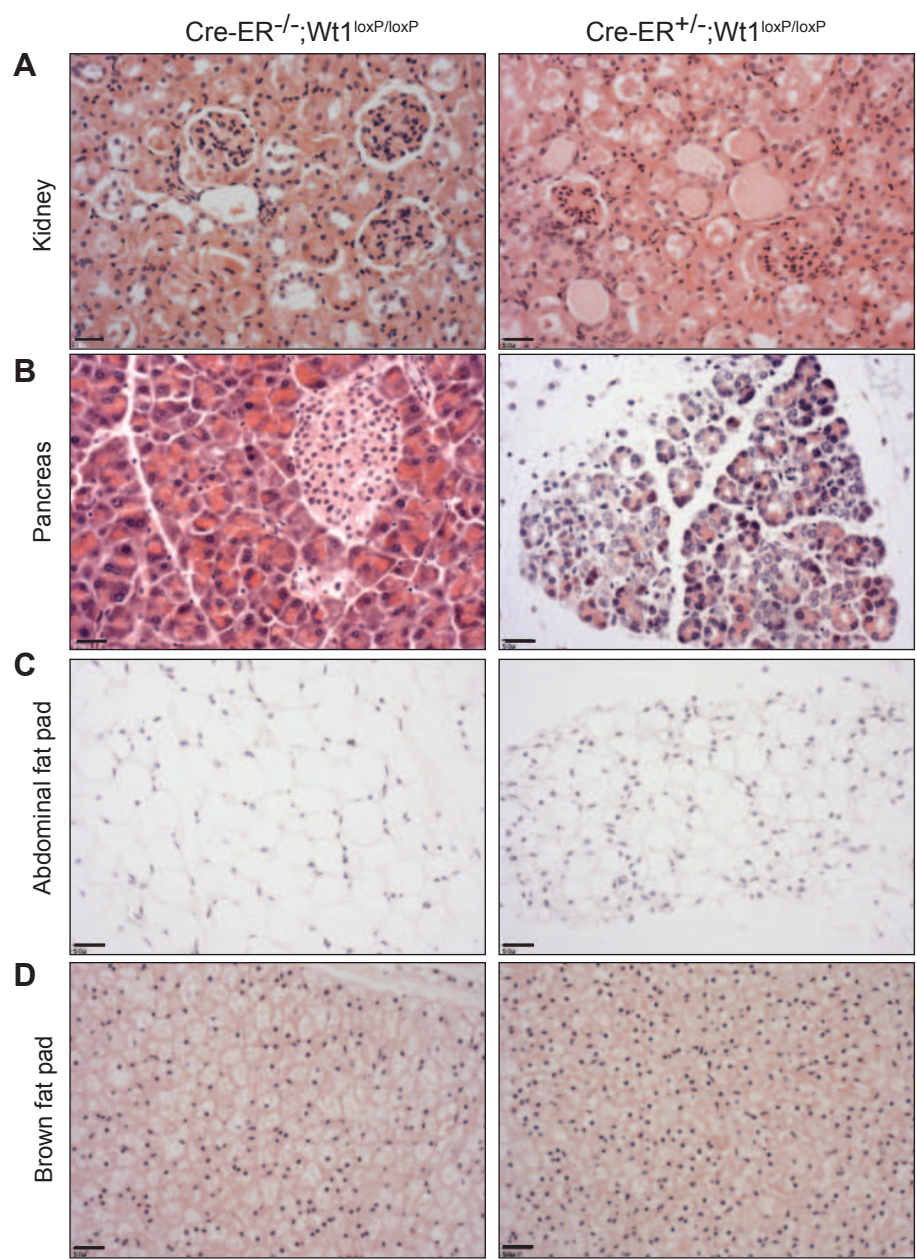

Supplement: Figure S4 — Characterisation of phenotypes in Wt1-KO mice at day 7 post-injection. H&E staining of sections from Wt1-KO mice. A, In the mutant kidney, protein casts are already visible. B, Moderate level of atrophy is seen in the mutant pancreas. C, The reduction in the size of fat vacuoles in the abdominal fat pad is already evident in the mutant mice. D, There is a slight reduction of the size of fat vacuoles in the brown fat pad from mutant mice; scale bars, 50 µm. (PDF) [file pgen.1002404.s004.pdf]

Figure S5

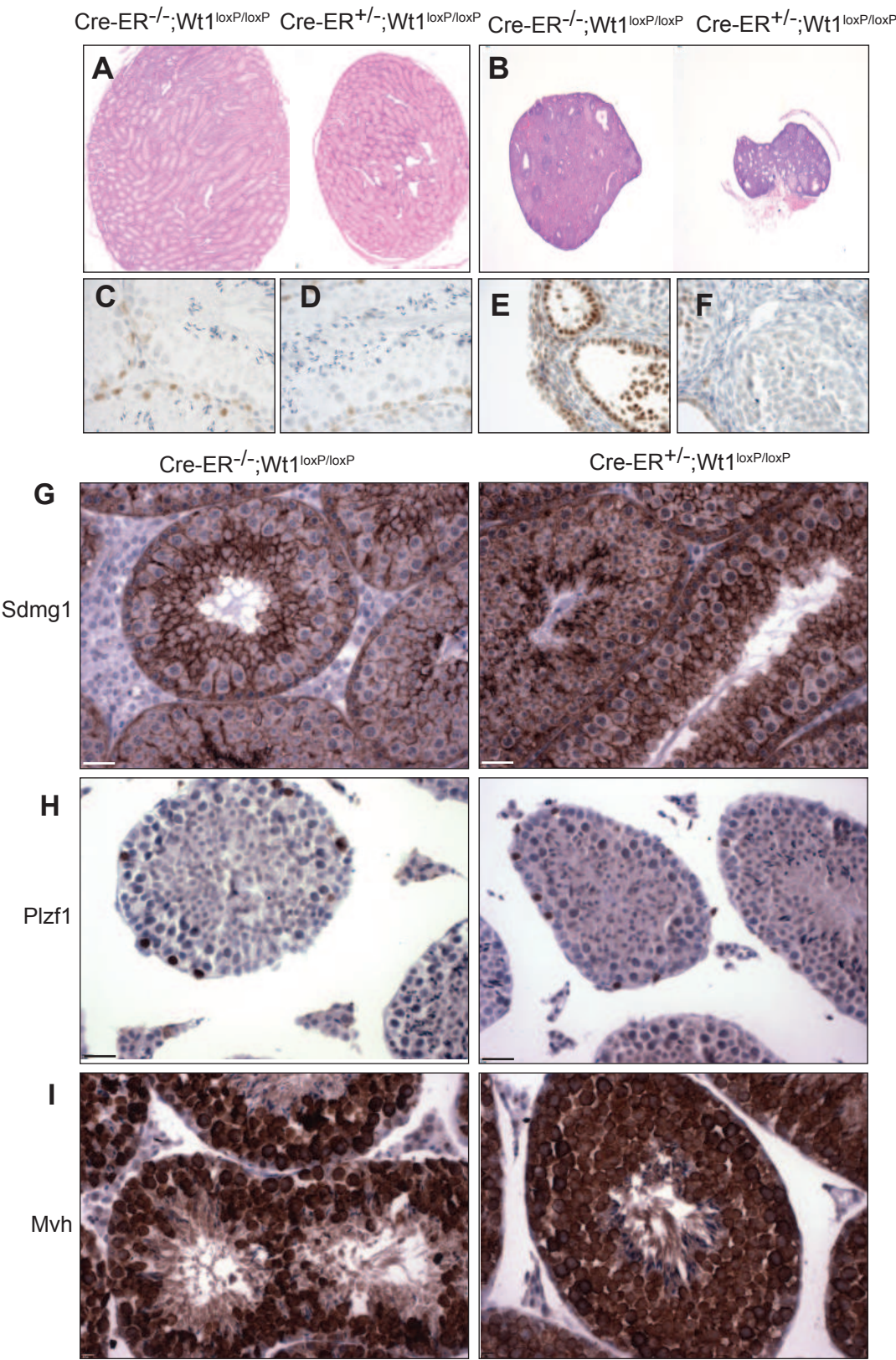

Supplement: Figure S5 — Minor gonadal defects in Wt1-KO mice. A, H&E staining show control (left) and mutant (right) testes. B, H&E staining of ovaries from control (left) and mutant mice (right). Follicles and corpora lutea are present in all mice but there was less luteal tissue in the mutant ovaries. In addition, there were fewer large, antral and atreic follicles in the mutant ovaries. Although the size of the gonads appear to be smaller in the mutants, the difference in the weights (e.g. testes) is not significant. Partial depletion of Wt1 expression in the Sertoli cells in the mutant testes (D) compared with the control (C). Wt1 staining in the granulosa cells in the ovaries (E) and its complete absence in the mutant (F). (G–I), Immunohistochemistry analysis of the expression of Sdmg1 (marker for Sertoli cells), Plzf1 (marker for spermatogonia), and Mvh (marker for spermatogonia, spermatocytes, and round spermatids) between control (left column) and mutant (right column) testes; scale bar, 50 µm. (PDF) [file pgen.1002404.s005.pdf]

Figure S6

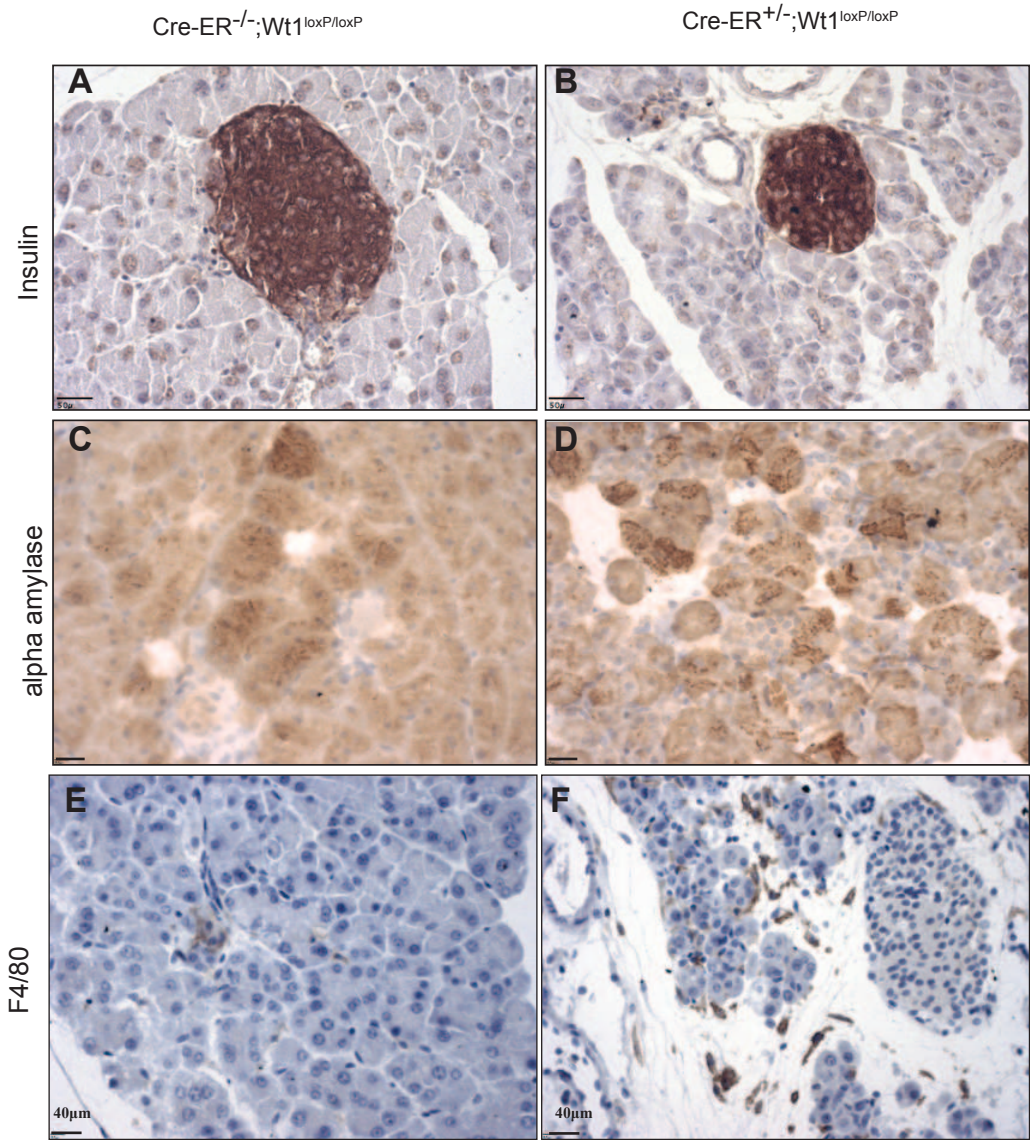

Supplement: Figure S6 — Immunohistochemistry analysis of markers in the pancreas. Images of sections from the mutant mice are shown in the right column and images from the control mice are shown in the left column. A–D, Immunohistochemistry staining indicate normal insulin and α-amylase expression in the mutant pancreas; scale bar, 50 µm. E, F, Using a pan marker for macrophages (F4/80), infiltrating macrophages are detected in the mutant pancreas; scale bar, 40 µm. (PDF) [file pgen.1002404.s006.pdf]

Figure S7

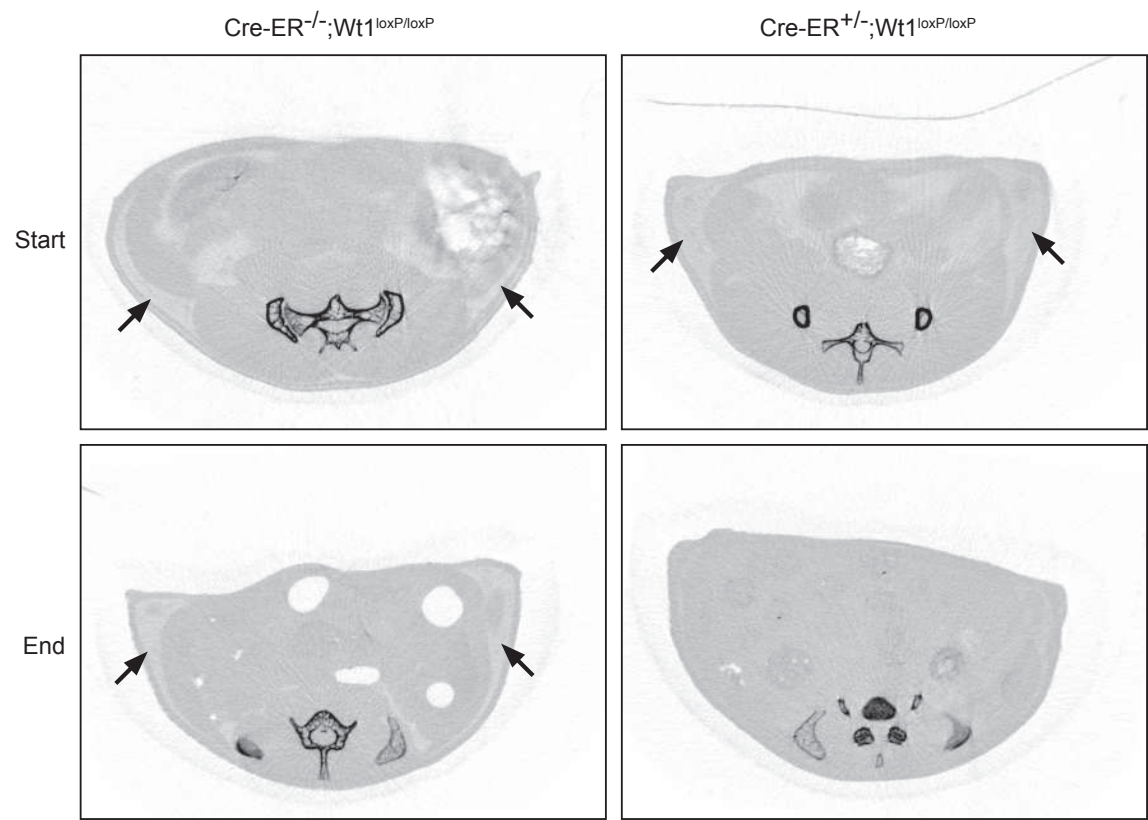

Supplement: Figure S7 — Fat reduction in mutant Wt1-KO using μCT. Representative transverse images taken from the μCT scanned mice before and after tamoxifen injection. Control mice (CreER™−/−, Wt1loxP/loxP) used for fat analysis are the sexed matched littermates of the mutant mice (CreER™+/−, Wt1loxP/loxP). Light grey shades indicate fat tissues which are present in both control and mutant mice before tamoxifen injection (arrows). Darker shades indicate soft tissues and black shades indicate skeletons. Gaps indicate gastric gas trapped in the intestines of the animal. After 9 days of tamoxifen injection, a reduction in the fat pads is noticed in the mutant mice. (PDF) [file pgen.1002404.s007.pdf]

Figure S8

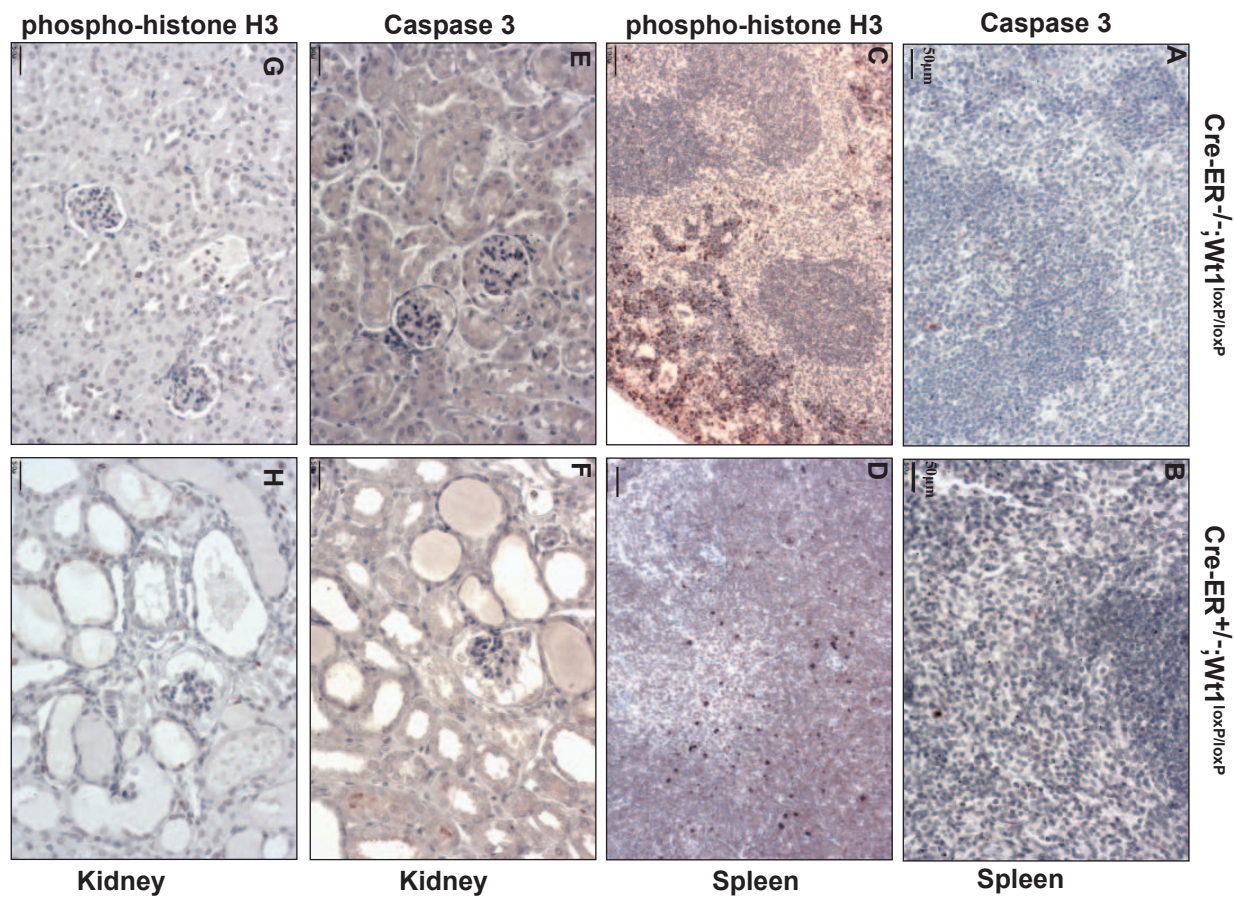

Supplement: Figure S8 — IHC staining of apotosis and proliferation markers in Wt1-KO mice. A,B, IHC staining of active caspase-3 in control (left) and mutant (right) spleen; scale bar = 50 um. C,D, IHC staining of phospho-histone H3 in control (left) and mutant spleen (right); scale bar = 100 um. E,F, IHC staining of active caspase-3 in control (left) and muatnt kidney (right); scale bar = 50 um. G,H, IHC staining of phospho-histone H3 in control (left) and mutant kidney (right); scale bar = 50 um. expression in the mutant pancreas; scale bar, 50 µm. (PDF) [file pgen.1002404.s008.pdf]
