# Supplementary material for: Acute Multiple Organ Failure in Adult Mice Deleted for the Developmental Regulator Wt1
Source: PLoS Genet. 2011 Dec 22;7(12):e1002404. doi: 10.1371/journal.pgen.1002404 (PMC3245305; doi:10.1371/journal.pgen.1002404)
Supplement: Table S1 — Antibodies and dilution factor. (PDF) [file pgen.1002404.s009.pdf]

Table S1 Antibodies and dilution factor

Antibodies	Company	Dilution
Wt1	GeneTex Inc	1:5000
Wt1	Santa Cruz	1:800-1:1600
Synaptopodin	Progen	1:1000
Nephrin	Sana Cruz	1:400
Ter119-PE	eBioscience	1:800
CD45-APC	eBioscience	1:800
CD11b-APC	eBioscience	1:600
Stro-1	R&D system	1:100
Caspase 3 (active)	R& D systems	1:1000
Ki67	Novocastra	1:2500
Desmin	Dako	1:800
Cytokeratin	Dako	1:200
Sdmg1	Ian Adam	1:200
Plzf1	Santa Cruz	1:200
Mvh	Abcam	1:200
Insulin	Sigma	1:1000-1:2000
Alpha amylase	Sigma	1:1000
F4/80	eBioscience	1:400
CD41- PE-Cy7	eBioscience	1:800
CD16/CD32-PE	eBioscience	1:400
Ter119- PE-Cy5.5	eBioscience	1:400
CD150-APC	Biolegend	1:100
c-kit- APC-eF780	eBioscience	1:1200
Sca-1-Pacific Blue	Biolegend	1:200
CD105-biotinylated	Biolegend	1:400
Streptavidin- PE-TxR	BD	1:800
CD4	BD	1:800
CD8a	BD	1:400
CD11b	eBioscience	1:100
Gr-1	BD	1:100
B220	BD	1:400
