# Supplementary material for: Acute Multiple Organ Failure in Adult Mice Deleted for the Developmental Regulator Wt1
Source: PLoS Genet. 2011 Dec 22;7(12):e1002404. doi: 10.1371/journal.pgen.1002404 (PMC3245305; doi:10.1371/journal.pgen.1002404)
Supplement: Table S2 — Sequences of primers and Roche Universal Probe Library number used for Q-PCR. (PDF) [file pgen.1002404.s010.pdf]

Table S2 Q-PCR primers and probes

Gene	Primers (5'-3')	Probe
aP2	gaaaacgagatggtgacaagc	#31
	gcccttcataaactcttgtgg	
Wt1	ggacgccctacagcagtg	#33
	catctgattccaggtcatgc	
18s rRNA	cgattggatggtttagtgagg	#81
	agttcgaccgtcttctcagc	
