# Supplementary material for: Acute Multiple Organ Failure in Adult Mice Deleted for the Developmental Regulator Wt1
Source: PLoS Genet. 2011 Dec 22;7(12):e1002404. doi: 10.1371/journal.pgen.1002404 (PMC3245305; doi:10.1371/journal.pgen.1002404)
Supplement: Text S1 — Supporting methods. (DOC) [file pgen.1002404.s011.doc]

**Text S1. Supporting methods**

**Histological analysis**

Mice were killed with carbon dioxide gas and weighed. Detailed postmortem examinations were conducted and gross abnormalities were noted. The following organs were weighed: heart, liver, kidneys (both), spleen, thymus, and testis (both). A standard phenotyping panel of organs and tissues were harvested for histopathological evaluation and fixed by immersion in 4% neutral buffered formalin for 72 hours. Organs and tissues for histopathology consisted of: skin (2 sites), lymphosalivary complex, gonads, stomach (forestomach, fundus and pylorus), small intestine (duodenum, jejunum, ileum), large intestine (caecum, transverse and descending colon), mesenteric lymph node, kidneys, lower urinary tract, uterus, coagulating gland and seminal vesicle, spleen, thymus, white fat depots (cervical, thoracic, abdominal, and inguinal pads, mesentery) infrascapular and periaortic brown fat, heart (base and ventricles), lungs, aorta, pancreas (head, body and tail), brain (coronal sections; Bregma 1.5, -1, and -6), endocrine organs (thyroid, adrenals, pituitary), head (3 coronal sections including eyes, middle and inner ears, and nasal mucosa), and femur-stifle joint-tibia and fibula. Bones were decalcified by immersion in 8% formic acid (Decalcifier I, Surgipath Europe, Ltd) for 24 hours. Fixed organs were embedded in paraffin, sectioned at 4 μm, and stained with hematoxylin and eosin.

**Immunohistochemistry**. Tissues were fixed and embedded in paraffin or OCT as previously described[5]. Information about antibodies used is available on request. Flourescent immunohistochemistry was performed on tissue sections using the protocol described previously[5], followed by incubation with Alexa Fluor conjugated secondary antibodies for one hour at room temperature. Slides were mounted using Vectorshield (Vector Laboratories) and nuclei were stained with DAPI.

**Fat vacuole size analysis**. Analysis of fat vacuoles was performed on the green colour coordinate of 24bit colour shading corrected images acquired with a 3.3 megapixel Qimaging micropublisher camera (40x objective). Weak membrane staining prevented automatic segmentation analysis. A script was written in IPLab (BioVision Inc) to provide interactive segmentation of the vacuoles followed by manual drawing of the incomplete membranes on the complementary segmentation mask by comparison with the original image. Vacuoles identified were outlined in a contrasting colour to provide a visual check, and data were accumulated in a text file, before transfer to Microsoft excel for further analysis.

**Recombination PCR**. Genomic DNA from tissues was extracted by digesting tissues overnight at 55ºC in Tail tip buffer (100 mM Tris.HCl pH8.0, 50 mM EDTA, 100 mM NaCl, and 1% SDS) containing Proteinase K (0.1mg/ml, Sigma). The debris was pelleted by centrifugation at 13K for 10 minutes. One ml of chilled 70% acetone/5% DMF was added to the supernatant and DNA was pelleted by centrifugation at 13K for 10 minutes. DNA pellet was washed in 70% ethanol on a rotator for two hours before dissolved in TE buffer. Primers used for detecting the Cre recombination are: WT1-Co/F (5’ tgggttccaaccgtaccaaaga 3’), Wt1-co/R1 (5’ gggcttatctcctcccatgt3’), and Wt1-co/R2 (5’ gtacgcgcgaacactgacta 3).

**Quantitative RT-PCR.** Primers were designed using the Ensembl Genome Browser and the Roche website (Roche; Burgess Hill, UK). Roche Universal Probe Library system probe and primers (Sigma) were used following the manufacturere’s instructions. Reactions were performed using either LightCycler 480 (Roche) or the 7900HT Fast Real-Time PCR System (Applied Biosystems). The reference gene used for all quantitative RT-PCR assays is 18s rRNA. Primer sequence and probe number is shown in Table S2.

**Serum and urine biochemical analysis**. Blood was collected by cardiac puncture and serum was harvested by centrifugation. Urine and serum biochemical analysis was performed by Dr Tertius Hough at MRC Harwell (UK).

**Cytokine quantification.** Levels of cytokine in mouse serum was tested using Proteome Profiler Mouse Cytokine Array Panel A Array Kit (RD Systems) and Proteome Profiler Mouse Adipokine Array Kit (RD Systems) according to the manufacturer’s protocol. Following washes to remove unbound material, the membranes were incubated with streptavidin DyLight 800 Conjugated antibody (Thermo Scientific, 1:5000) for 30 minutes at room temperature. The signals were quantified using Odyssey Sa Infrared Imaging System.

**Mouse growth hormone (GH) ELISA.** Level of growth hormone (GH) in mouse serum was tested using ELISA (Millipore, MA) following the manufacturer’s protocol.

**Flow cytometry analysis**. Fluorescence activated cell sorting (FACS) analysis was performed on bone marrow and splenocytes. Lineage markers (CD45-FITC, CD45-APC, CD11b-APC, GR1-FITC, and TER119-PE) were purchased from BD Biosciences (San Jose, CA) or e-Bioscience (San Diego, CA). For myeloerythroid progenitor staging, spleen was made into single cell suspension and stained with lineage antibodies (CD4, CD8, CD11b, Gr1 and B220) and antibodies against Sca1, c-kit, FcgR, CD41, CD150 and CD105. 7-Amino-Actinomycin (7-AAD) was used to exclude dead cells. Antibody dilution and suppliers are indicated in Table S1. For multi colour analysis and sorting, colour compensation samples were produced by singly staining spleen cells with one antibody of each of the combined fluorochromes. FACS analysis and sorting were performed using a FACS Flow Cytometer and data were analysed using FlowJo software.

**Methylcellulose colony formation assays.** Bone marrow from tamoxifen treated [*CAGG-CreER*^TM^, *Wt1*^loxP/loxP^] mice were harvest at day 10 post-injection and resuspended in IMDM(+10% FCS). 200x10^3^ cells were mixed thoroughly in 400 μl methylcellulose haematopoietic colony assay medium (MethoCult H4434, StemCell Technologies). Cell mixtures were plated in 24-well plate and cultured for up to two weeks. Haematopoietic cell colonies were visualised using a phase contrast microscope and pooled in PBS for flow cytometry analysis.

**Enrichment and culturing of pancreatic and hepatic stellate cells**. Pancreas and liver from 7 adult mice were collected and digested in HBSS buffer containing pronase (100mg, Sigma Fluka), collagenase B (20mg, Roche), and DNase (10mg, Roche) for 20 minutes at 37ºC. The suspension was scrapped through a mesh and pelleted by centrifuging at 600 rcf for 7 minutes. The stellate cells were enriched and cultured by using the Optiprep gradient as described in previous study (Shek F *et al* 2002).

**Bone marrow adipocyte quantification**. Total bone marrow cells were flushed out from the tibia and femurs of [*CAGG-CreER*^TM^, *Wt1*^loxP/loxP^] mice in α-MEM supplemented with 10% FCS and 10μg/ml insulin). AdipoRed (Lonza Ltd) was used label adipocytes and the number of adipocyte was quantified using FACS analysis.

**Mouse osteoclast cultures and tartrate resistant acid phosphatase (TRAcP) assay.** Osteoclasts were generated from bone marrow cells as described in Idris A *et al* (2009). Osteoclasts were identified by tartrate resistant acid phosphatase (TRAcP) staining ad was performed as described in R. van’t Hof (2008). Cells were considered to be osteoclast if they were TRAcP positive and contained three or more nuclei.

**Mouse osteoblast cultures and alkaline phosphatase assay.** Osteoblast cells were generated from bone marrow cells as described in Idris A *et al* (2009). After culturing for approximately 10 days, 4-OH tamoxifen was added in the culture medium (1μM) for three days to induce *Wt1* deletion. Adherent cells were harvested by trypsin digestion and plated in 96-well plates at 10^4^ cells per well, cultured for an additional 48 h. Cell number was determined using Alamar blue assay and alkaline phosphatase (ALP) activity was measured as described in Idris A *et al* (2009).

**Bone density and bone histomorphometry.** Bones were dissected free from soft tissue and fixed overnight in neutral buffered formalin overnight, and stored in 70% ethanol. Bone architecture was determined by µCT analysis using a Skyscan 1172 system at a resolution of 5 µm (60 kV, 167 µA). Scans were reconstructed using the Skyscan NRecon software and bone parameters measured at the distal femur, proximal tibia and fifth lumbar spine using Skyscan CTAn software. After the µCT analysis, the bones were decalcified in buffered EDTA and embedded in glycol methacrylate. Five µm sections were stained for TRAcP as described above and counterstained with haematoxylin.

**Statistical analysis.** Results are expressed as mean ± s.e.m. Data were analysed by Mann-Whitney Test using SPSS statistical program. *P* values <0.05 were considered significant. Error bars depict s.e.m.

Shek FW, Benyon RC, Walker FM, McCrudden PR, Pender SL, et al. (2002) Expression of transforming growth factor-beta 1 by pancreatic stellate cells and its implications for matrix secretion and turnover in chronic pancreatitis. Am J Pathol 160: 1787-1798.

Idris AI, Sophocleous A, Landao-Bassonga E, Canals M, Milligan G, et al. (2009) Cannabinoid receptor type 1 protects against age-related osteoporosis by regulating osteoblast and adipocyte differentiation in marrow stromal cells. Cell Metab 10: 139-147.

van’t Hof R (2008) Bone Research Protocols: Humana Press.
